# Evaluation of vector systems and promoters for overexpression of the acarbose biosynthesis gene *acbC* in *Actinoplanes* sp. SE50/110

**DOI:** 10.1186/s12934-019-1162-5

**Published:** 2019-06-28

**Authors:** Lena Schaffert, Camilla März, Lisa Burkhardt, Julian Droste, David Brandt, Tobias Busche, Winfried Rosen, Susanne Schneiker-Bekel, Marcus Persicke, Alfred Pühler, Jörn Kalinowski

**Affiliations:** 1 Microbial Genomics and Biotechnology, Center for Biotechnology, Bielefeld University, Universitätsstraße 27, 33615 Bielefeld, Germany; 2 Senior Research Group in Genome Research of Industrial Microorganisms, Center for Biotechnology, Bielefeld University, Universitätsstraße 27, 33615 Bielefeld, Germany; 3 Product Supply, Bayer AG, Friedrich Ebert Str. 217-475, 42117 Wuppertal, Germany

**Keywords:** *Actinoplanes*, Acarbose, pKC1139, pSET152, Promoter screening, *gusA*, TSS detection

## Abstract

**Background:**

*Actinoplanes* sp. SE50/110 is a natural producer of acarbose. It has been extensively studied in the last decades, which has led to the comprehensive analysis of the whole genome, transcriptome and proteome. First genetic and microbial techniques have been successfully established allowing targeted genome editing by CRISPR/Cas9 and conjugal transfer. Still, a suitable system for the overexpression of singular genes does not exist for *Actinoplanes* sp. SE50/110. Here, we discuss, test and analyze different strategies by the example of the acarbose biosynthesis gene *acbC*.

**Results:**

The integrative φC31-based vector pSET152 was chosen for the development of an expression system, as for the replicative pSG5-based vector pKC1139 unwanted vector integration by homologous recombination was observed. Since simple gene duplication by pSET152 integration under control of native promoters appeared to be insufficient for overexpression, a promoter screening experiment was carried out. We analyzed promoter strengths of five native and seven heterologous promoters using transcriptional fusion with the *gusA* gene and glucuronidase assays as well as reverse transcription quantitative PCR (RT-qPCR). Additionally, we mapped transcription starts and identified the promoter sequence motifs by 5′-RNAseq experiments. Promoters with medium to strong expression were included into the pSET152-system, leading to an overexpression of the *acbC* gene. AcbC catalyzes the first step of acarbose biosynthesis and connects primary to secondary metabolism. By overexpression, the acarbose formation was not enhanced, but slightly reduced in case of strongest overexpression. We assume either disturbance of substrate channeling or a negative feed-back inhibition by one of the intermediates, which accumulates in the *acbC*-overexpression mutant. According to LC–MS-analysis, we conclude, that this intermediate is valienol-7P. This points to a bottleneck in later steps of acarbose biosynthesis.

**Conclusion:**

Development of an overexpression system for *Actinoplanes* sp. SE50/110 is an important step for future metabolic engineering. This system will help altering transcript amounts of singular genes, that can be used to unclench metabolic bottlenecks and to redirect metabolic resources. Furthermore, an essential tool is provided, that can be transferred to other subspecies of *Actinoplanes* and industrially relevant derivatives.

**Electronic supplementary material:**

The online version of this article (10.1186/s12934-019-1162-5) contains supplementary material, which is available to authorized users.

## Background

The slowly growing, spore-forming, Gram-positive bacterium *Actinoplanes* sp. SE50/110 (ATCC 31044), is a natural derivative of SE50. It was isolated from a soil sample during a screening program by the Bayer AG in 1970 as natural producer of an α-glucosidase inhibitor [1, 2]. The discovered inhibitor, subsequently known as acarbose, consists of the pseudo-tetrasaccharide acarviosyl-1,2-maltose, which leads to the irreversible inhibition of α-glucosidases, like the one from the human intestine [3]. Physiologically, the inhibition of intestinal glucosidases leads to a retarded release of monosaccharides, especially of glucose, and therefore reduced resorption and decreased postprandial blood and serum sugar levels. These are assumed to be crucial for the cardiovascular disease mortality in the context of the complex pathology of diabetes [4, 5]. Since the early 1990s acarbose is used in the medical treatment of type II diabetes mellitus and marketed under the name Glucobay^®^ by the Bayer AG [4, 6].

**Fig. 1:**
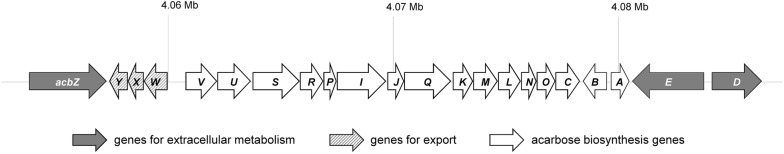
The acarbose biosynthesis gene cluster and gene disposition in the genome of *Actinoplanes* sp. SE50/110 (GenBank: LT827010.1)

**Fig. 2:**
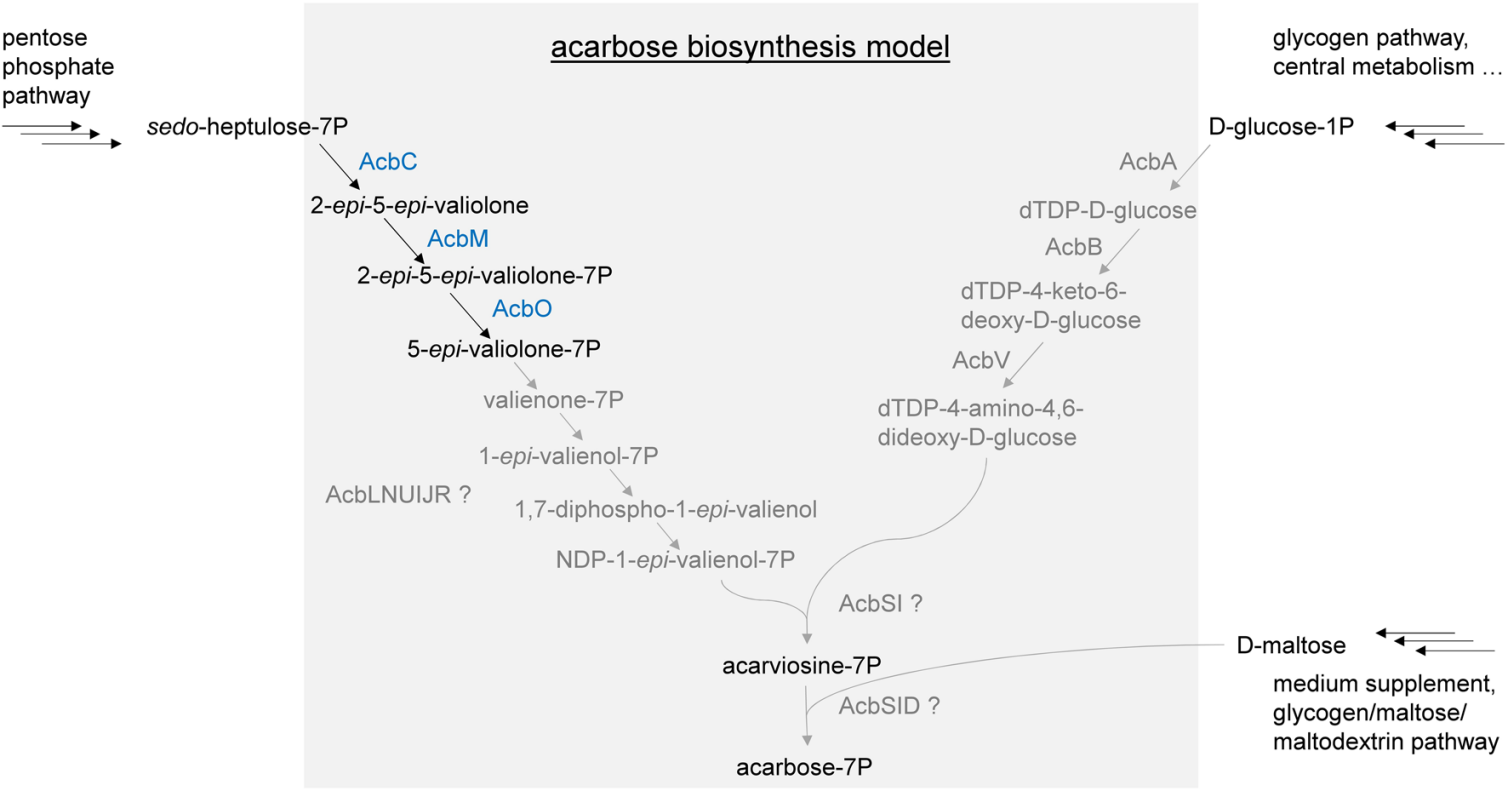
Current model of acarbose biosynthesis according to protein homologies and functional predictions [6, 11, 13]. The first three steps, catalyzed by AcbC, AcbM and AcbO (shown in blue), were experimentally proven [7, 11, 12]

The biosynthetic pathway of aminoglycosides—like acarbose—is based on monofunctional enzymes catalyzing single steps [3]. Their corresponding biosynthesis gene cluster was first identified in 1999 by Stratmann et al. and subsequently sequenced (GenBank: Y18523.4) [7, 8]. The cluster contains 22 genes (Fig. 1), including genes predicted to encode for proteins of the biosynthetic pathway (AcbCMOLNUJRSIVBA), extracellular starch degradation (AcbEZ) and transglycosylation (AcbD), export and subsequent dephosphorylation of acarbose (AcbWXY), and furthermore for an acarbose-7-kinase (AcbK) and an intracellular amylomaltase (AcbQ) [9, 10]. Except of the first three steps of acarbose biosynthesis, which were experimentally proven [7, 11, 12], the recent model of acarbose biosynthesis is based on protein homologies and functional predictions [6, 11, 13] (Fig. 2). AcbC, the first enzyme of acarbose biosynthesis, catalyzes a cycling reaction to generate 2-*epi*-*5*-*epi*-valiolone from *sedo*-heptulose-7P [7]. As *sedo*-heptulose-7P is derived from the pentose phosphate pathway, AcbC catalyzes the transition from the primary to the secondary metabolism [12].

In the last decades the acarbose producer *Actinoplanes* sp. SE50/110 became a focus of research and the complete genome [10], transcriptome [14] and proteome [9, 15] were analyzed comprehensively. This led to a refined genome sequence and annotation in 2017 (GenBank: LT827010.1) [16].

By knowledge of *omics* data and establishing of an intergeneric conjugation system [17] as well as advanced genome editing tools by use of CRISPR/Cas9 (clustered regularly interspaced short palindromic repeats/CRISPR-associated endonuclease 9) [18], fundamental prerequisites for the future strain development by targeted genetic engineering have been fulfilled. Still, a reliable expression system allowing medium to strong gene expression in *Actinoplanes* sp. SE50/110 is needed. A lot of applications of such system exist, f. e. the redirection of metabolic resources, removal of metabolic bottlenecks and/or the unraveling of genetic functions.

We analyzed two vector systems, a replicative and an integrative system. Moreover, the strength of several promoters was tested by use of the *gusA* reporter system developed by Horbal et al. [19]. These promoters were used to overexpress the *acbC* gene on the integrative vector. By development of an overexpression system for *Actinoplanes* sp. SE50/110 and similar species an important step for future work in identifying putative metabolic bottlenecks is taken.

## Results and discussion

### Unintended chromosomal integration of pKC1139-based vectors by homologous recombination in *Actinoplanes* sp. SE50/110

The replicative vector pKC1139, constructed by Bierman et al. [20], is a pOJ260-derivative with temperature-sensitive pSG5 replicon from *Streptomyces ghananensis* (taken from the plasmid pSW344E [21]), which allows replication at temperatures below 34 °C in various Actinomycetales.

The vector has been successfully used as an expression vector in the closely related teicoplanin producer *Actinoplanes teichomyceticus* [22]. Also transformation into *Actinoplanes* sp. SE50/110 displays high transformation efficiency as well as high vector stability—even in case of exposure to an elevated temperature of 37 °C [23]. This indicates, that this vector system might be beneficial as expression system.

Other replicative *Streptomyces*–*E. coli* shuttle plasmids, like the SCP2*-replicon based pKC1218 [24] and the pIJ101-replicon based pSOK101 [25], did not give exconjugants with *Actinoplanes* sp. SE50/110 [23]. These replicons are probably unstable or inactive in *Actinoplanes* sp. SE50/110, which is also in accordance with findings from the related species *A. teichomyceticus* [22].

Due to this, pKC1139 was chosen as replicative expression plasmid and tested in *Actinoplanes* sp. SE50/110 for the individual expression of eight *acb* genes (*acbR* (*ACSP50_3597*), *acbQ* (*ACSP50_3601*), *acbK* (*ACSP50_3602*), *acbM* (*ACSP50_3603*), *acbL* (*ACSP50_3604*), *acbN* (*ACSP50_3605)*, *acbO* (*ACSP50_3606*), *acbC* (*ACSP50_3607*)), under control of the ermE*-promoter from *Saccharopolyspora erythraea*, which has shown to be active in *Actinoplanes* sp. SE50/110 [23].

Exconjugants of *Actinoplanes* sp. SE50/110 were proven by polymerase chain reaction (PCR) for the presence of the vector pKC1139 carrying the respective *acb* gene and afterwards cultivated in maltose minimal medium. In all cases, the pKC1139-mutants grew normally (data not shown). In case of pKC1139-mutants of *acbR*, *acbK*, *acbL*, *acbM*, *acbN* and *acbO* no acarbose formation was detected. In the case of *acbQ* a strongly reduced amount of acarbose was found, whereas pKC1139-mutants of *acbC* produced at the wild-type level (data not shown).

**Fig. 3:**
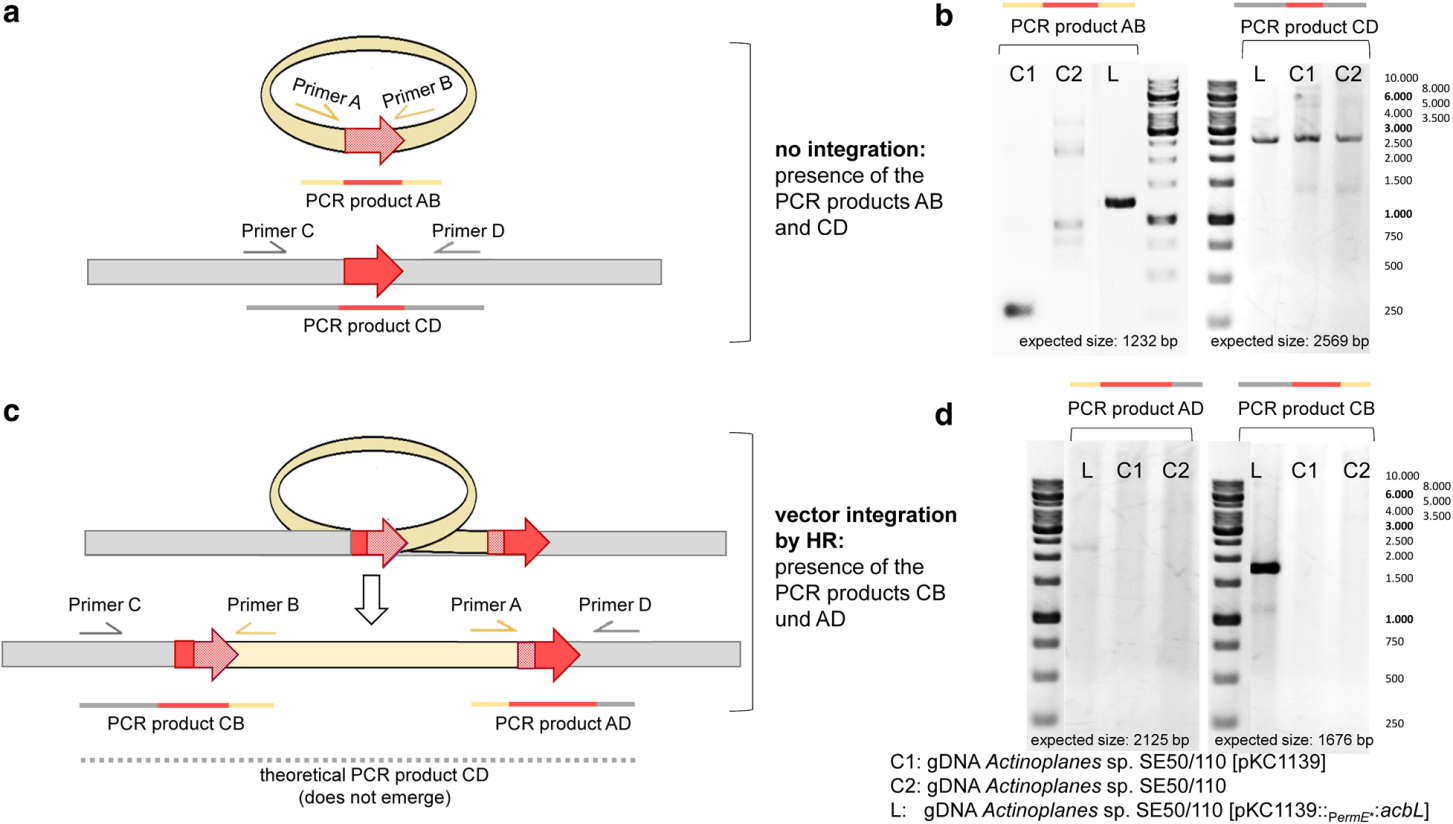
PCR from genomic DNA (gDNA) to prove vector integration of pKC1139-based constructs by homologous recombination. **a** Scheme of tested primer combinations and expected PCR products in case of no vector integration: Primer A and B form PCR-product AB indicating presence of the insert. Primer C and D form PCR-product CD flanking the genomic location of the gene of interest. Both PCR-products occur in the mutant *Actinoplanes* sp. SE50/110 [pKC1139:: P_ermE*_::::*acbL*] (L) (**b**). **c** Scheme of tested primer combinations and expected PCR products in case of vector integration by homologous recombination (HR) leading to the PCR-products CB (from Primer C and B) and AD (from Primer A and D). Both PCR-products occur in the tested mutant *Actinoplanes* sp. SE50/110 [pKC1139::P_ermE*_::*acbL*] (L) indicating, that vector integration by homologous recombination has occurred (**d**). The primer combination AB does not form a PCR product in this constellation. Primer combination CD might theoretically produce a larger product (grey dashed lines), but due to the size, GC-content and complex structures within the vector sequence leading to premature termination of the polymerase reaction, this product does not emerge during PCR. As control for all PCR experiments the empty vector control *Actinoplanes* sp. SE50/110 [pKC1139] (C1) and the wild-type (C2) were tested. Here, only primer combination C and D led to the expected fragment CD (**b**)

As the loss of acarbose production was surprising, all mutants were analyzed in more detail. A PCR experiment was designed, which tests for vector integration by homologous recombination (HR). Such event might occur by single cross-over between the two gene copies, one of which is localized on the vector and the second in the genome. Primers were designed, binding adjacent to the gene of interest either within the vector region (for testing of the vector-insert) or binding adjacent to the locus of the gene of interest (for testing of the intactness of the genomic locus) (Fig. 3a). By combination of one PCR primer binding on the vector and the other binding adjacent to the genetic locus, it is possible to detect vector integration, as this primer combination can only lead to a distinct PCR product, when this event has occurred (Fig. 3c). Indeed, integration of the vector into the locus of the gene of interest was detected in all cases (Fig. 3d, Additional file 1: Data S1). In the complex cell sample used for genomic DNA (gDNA)-isolation, also cells without vector integration exist, which was shown as well by PCR (Fig. 3b, Additional file 1: Data S1).

It has to be noted, that the empty vector was maintained without antibiotic pressure, which was proven by parallel plating on antibiotic-containing solid medium after several days of antibiotic-free cultivation, like it has already been shown by Gren [23]. As the empty vector cannot be integrated into the host genome by HR, maintenance of the vector by pSG5-replication is assumed.

By the single-crossover directing the vector integration, the *acb* gene cluster is disrupted, probably leading to premature termination or even degradation of the polycistronic mRNA (messenger RNA) and loss of acarbose production in six cases (*acbRQKMNLO*). Such negative impact on the expression of genes located downstream of the target gene was observed by reverse transcription quantitative PCR (RT-qPCR) [exemplary shown for *Actinoplanes* sp. SE50/110 [pKC1139::*P*_ermE*_::*acbL*] (Additional file 1: Data S2)].

In the case of the gene *acbC*, which is localized at the end of the *acb* gene cluster (compare to Fig. 1), the expression of further *acb* genes is not influenced by vector integration. Also, the *acbC* gene copies remain intact during the recombination process. Therefore, it is conclusive that this mutant is still able to produce acarbose, whereas in all the other pKC1139-mutants acarbose formation was abolished or extremely reduced.

Homologous recombination is a common process in Actinobacteria: in various *Streptomyces* this process is utilized to create deletion mutants by double-crossover. Temperature-sensitive replicons, like the pSG5-replicon, can support and force this process in *Streptomyces* ssp. [26–29]. In contrast, previous studies indicated successful expression of homologous genes in the related species *A. teichomyceticus* [22]. In this work, we could show that expression vectors carrying homologous regions tend to be integrated into the corresponding genetic locus of the host SE50/110. BLAST analysis against the NCBI-database [30] lead to the identification of *ACSP50_7170*, predicted as recombinase A gene (*recA*), which might catalyze such kind of recombination process. Furthermore, no homolog was found in the genome of *A. teichomyceticus* (not shown), which might explain, why such process has not been reported for this species before.

In contrast to *A. teichomyceticus* and although high vector stability has already been shown for the empty vector in SE50/110, the replicative pSG5-based vector pKC1139 is not suitable as expression vector in *Actinoplanes* sp. SE50/110, as vector integration by homologous replication seems to be a favored process, putatively due to the metabolic costs of vector replication. A pSG5-based expression system might be implemented in future by deletion of the recombinase gene *recA*.

### Gene duplication by use of the integrative pSET152 vector system did not lead to enhanced expression of genes

Consequently, integrative vector systems were tested as vehicles for the expression of homologous genes in *Actinoplanes* sp. SE50/110.

Four different integrative vectors have already been described for *Actinoplanes* sp. SE50/110 [17]: Two are based on the integration mechanism of φC31 (pSET152 and pIJ6902), which is well studied among related Actinomycetales, one is based on the integration mechanism of φBT1 (pRT801) and one on the VWB-phage integration mechanism (pSOK804) [17]. For implementing of an expression system in *Actinoplanes* sp. SE50/110, the vector pSET152 was chosen, as it is best studied and broadly used in Actinomycetales (GenBank: AJ414670.1). Additionally, it had been recently reported to be used for the expression of a homologous gene under control of an heterologous promoter in *Actinoplanes* sp. SE50/110 [31].

**Fig. 4:**
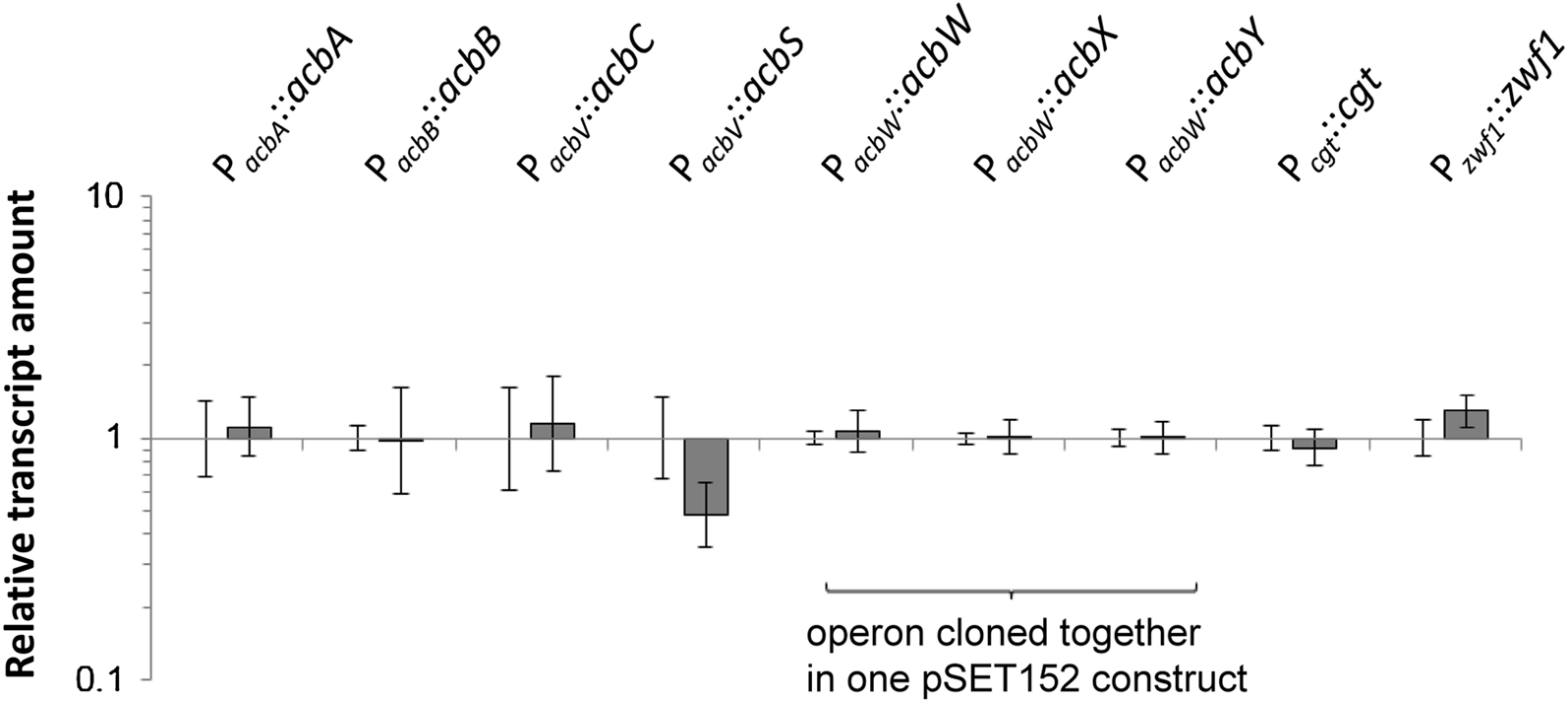
Relative transcript amounts of single genes in different pSET152-mutants of *Actinoplanes* sp. SE50/110. The genes *acbA*, *acbB*, *acbWXY*, *zwf* and *cgt* were transcribed under control of their native promoter. In case of *acbC* and *acbS* the promoter upstream of *acbV* was used, which is the first gene of the respective operon. The RNA was isolated from triplicates of the growth phase of a shake flask cultivation in maltose minimal medium and analyzed by RT-qPCR. The relative transcript amounts were analyzed in relation to the amounts of the empty vector control *Actinoplanes* sp. SE50/110 [pSET152] (relative transcript amount set to 1)

In initial experiments, seven different pSET152 constructs were transferred to *Actinoplanes* sp. SE50/110 by conjugation to achieve a gene duplication: Five of which were carrying genes of the *acb* gene cluster (*acbA* (*ACSP50_3609*), *acbB* (*ACSP50_3608*), *acbC* (*ACSP50_3607*), *acbS* (*ACSP50_3596*), *acbWXY* (*ACSP50_3591*-*3*)), one carrying a gene of central metabolism *zwf (ACSP50_1790)* encoding for a glucose-6-phosphate dehydrogenase, and another construct carrying the gene *cgt* (*ACSP50_5024*), which has shown to be strongly transcribed and translated in *Actinoplanes* sp. SE50/110 and encodes a small carbohydrate binding protein [13, 14]. The genes *zwf* and *cgt* were selected as controls representing a medium and a strong promoter. All genes were controlled by their own native promoters. In the case of *acbC* and *acbS*, the promoter upstream of the first gene of the respective operon was used [here: the promoter in front of *acbV* (*ACSP50_3594*)]. Exconjugants were grown in maltose minimal medium displaying normal growing and acarbose producing phenotypes compared to the empty vector control *Actinoplanes* sp. SE50/110 [pSET152] and the wild-type (data not shown). Interestingly, RT-qPCR experiments showed, that no duplication of transcript amount was achieved for each of these constructs compared to the empty vector control (Fig. 4). Similar findings have already been obtained by Wolf et al. [32]. Here, pSET152-based expression by use of a native promoter was used for complementation of a deletion mutant, leading to only half of the transcript amount compared to the wild-type [32].

This might be caused by nonspecific effects, like f. e. by deletion of the gene *ACSP50_6589* (former: *ACPL_6602*), which carries the φC31-integration site, by influencing of the transcription of the genes in direct vicinity to the integration site, or by integration of the vector into secondary (pseudo)-integration sites.

*ACSP50_6589* is annotated as hypothetical pirin-homologue [17]. In mammals, pirin is a transcriptional cofactor related to apoptosis-proteins with unknown function [33]. In bacteria it had been hypothesized to influence biological and phenotypical processes, but the exact function was not understood [34, 35]. As φC31-integration vectors like pSET152 are broadly used in Actinobacteria, disruption of the pirin-homolog became a focus of scientific discussion: In some species of the order Actinomycetales reduced [36–38] respectively improved [22] antibiotic production was shown, whereas for some species no aberrant growth or producing phenotype have been reported [36].

Previous reports from *Actinoplanes* SE50/110 [17, 31] attest normal viability and acarbose production of pSET152-integration mutants under laboratory conditions. This was also confirmed by our work (data not shown).

By RNAseq of two pSET152-integration mutants no direct effects on the transcription of the genes in direct vicinity were found (data not shown).

Besides, no additional integration site or genomic rearrangements were detected in *Actinoplanes* sp. SE50/110 [17, 23], like it had been reported before for species of the family Streptomyces [39, 40] and *Saccharopolyspora spinosa* [41].

For this reason, we could not observe any nonspecific effects in SE50/110 by pSET152-integration. As by use of native promotors, a doubling of relative transcript amounts was not achieved, a strict regulation of native promoters by the regulatory network of *Actinoplanes* sp. SE50/110 is assumed.

Here, we address this problem by integration of strong promotors—especially heterologous promotors beyond the cellular control—to achieve overexpression of the gene of interest in *Actinoplanes* sp. SE50/110.

### Evaluation of heterologous and homologous promoters by screening experiments

For the development of a pSET152-based expression system, a promoter screening was carried out to find valuable candidates for the overexpression of the *acb* genes. For this purpose the screening system of Horbal et al. [19] was used, which is based on the reporter GusA, a glucuronidase, which is able to hydrolyze 5-bromo-4-chloro-3-indolyl glucuronide (X-Gluc). Two molecules of 5-bromo-4-chloro-3-indoxyl congregate to 5,5′-dibromo-4,4′-dichloro-indigo, which is a blue pigment. The reporter gene is cloned on a pSET152-backbone. Table 1 shows the tested heterologous and homologous promoters, of which four were provided by the working group of Andriy Luzhetskyy (Saarland University, Saarbrücken, Germany).

**Table 1:** Constructs with the reporter gene *gusA* tested in this promoter screening experiment

	Abbr.	Origin of promoter	Construct	Source/comment
Homologous	*cgt*	Promoter of *cgt* (*ACSP50_5024*), annotated as small carbohydrate binding protein	pSET*cgt*P*gusA*	This work
	*efp*	Promoter of *efp* (*ACSP50_6465*), annotated as the translation elongation factor P	pSET*efp*P*gusA*	This work
	*7457*	Promoter of *ACSP50_7457*, annotated as hypothetical protein	pSET*7457*P*gusA*	This work
	*katE*	Promoter of *katE* (*ACSP50*_*3066*), annotated as catalase hydroperoxidase (HP) II	pSET*katE*P*gusA*	This work
	*rpsJ*	Promoter of *rpsJ* (*ACSP50*_*0690*), annotated as 30S ribosomal protein S10	pSET*rpsJ*P*gusA*	This work
Heterologous	*tipA*	Promoter of *tipA* from *S. lividans*, annotated as HTH-type transcriptional activator [42]	pSETGUS	Myronovskyi et al. [43]
	*moeE5*	Promoter of *moeE5* from *S*. *ghanaensis*, a central moenomycin A biosynthetic gene encoding for a nucleotide sugar epimerase [44]	pSETPmoeE5	Horbal et al. [19] and R. Makitrynskyy, Ivan Franko National University, Lviv, Ukraine
	*apm*	Promoter of *aac(3)IV* from pCRISPomyces-2 [45], an aminoglycoside 3-*N*-acetyltransferase, which mediates apramycin resistance	pSET*aac(3)IV*P*gusA*	This work
	*cdaR*	Promoter of *cdaR* from *S. coelicolor*, encoding for a transcriptional regulator (proposed as activator of a calcium-dependent antibiotic CDA [46, 47])	pSETPcdaRgusA	Horbal et al. [19]
	*actP*	Promoter of *actII*-*4* from *S. coelicolor*, annotated as actinorhodin operon activator protein [48]	pSET*act*P*gusA*	This work, using primers and design from Horbal et al. (2013) [19]
	ermE*	Promoter of ermE* from *S. erythraea*, annotated as 23S rRNA (adenine-N6)-dimethyltransferase mediating erythromycin resistance [49, 50]	pGUSPErmE	This work, referring to Siegl et al. [51]; Bibb et al. [52]
	*gapDH*	Promoter of *gapDH* from *Eggerthella lenta* used on pCRISPomyces-2 [45], annotated as type I glyceraldehyde-3-phosphate dehydrogenase	pSET*gapDH*P*gusA*	This work
	*rpsL*	Promoter of *rpsL* from *Xylanimonas cellulosilytica* used on pCRISPomyces-2 [45], annotated as ribosomal protein S12	pSET*rpsL*P*gusA*	This work
	pGUS	No promoter	pGUS	Myronovskyi et al. [43]

**Fig. 5:**
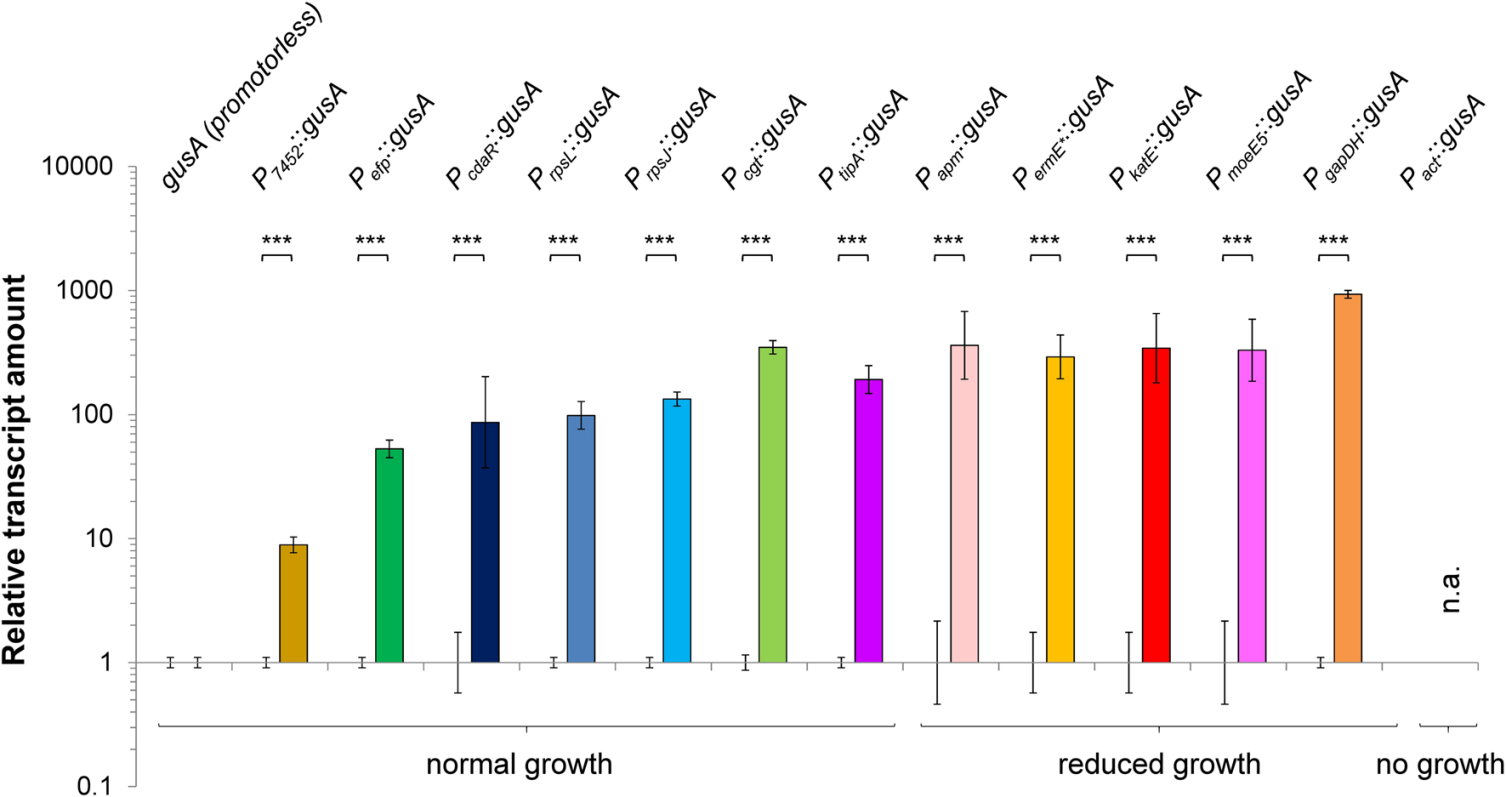
Relative transcript amounts of the *gusA* gene under control of different homologous and heterologous promoters in *Actinoplanes* sp. SE50/110. The RNA was isolated from the growth phase of a shake flask cultivation in maltose minimal medium and analyzed by RT-qPCR. The calculated averages and standard deviations of a minimum of three biological replicates are shown. The transcription amounts of *gusA* gene are analyzed in relation to the amounts of the *Actinoplanes* sp. SE50/110 mutant carrying the promoterless pGUS-vector (relative transcript amount of promoterless pGUS set to 1). For *actP* no RNA could be isolated due to a severe growth deficiency in maltose minimal medium. (p-values of a two-sided t-test: P_*2475*_: 0.0001133, P_*efp*_: 4.871e−05, P_*cdaR*_: 0.002509, P_*rpsL*_: 9.928e−06, P_*rpsJ*_: 1.167e−08, P_*cgt*_: 5.911e−08, P_*tipA*_: 7.158e−06, P_*apm*_: 4.596e−05, P_ermE*_: 0.0009364, P_*katE*_: 0.0001373, P_*moeE5*_: 0.0002518, P_*gapDH*_: 4.207e−06)

Strains carrying 13 different promoter constructs were grown in maltose minimal medium. Promoter strengths were monitored by glucuronidase assays during early until late growth phase (Additional file 1: Data S3) and on transcriptional level by RT-qPCR (Fig. 5). Both—GUS assay and RT-qPCR—display similar tendencies, which allows categorizing the promoters into weak (*7457*), medium (*efp*, *cdaR*, *rpsL*, *rpsJ*, *cgt* and *tipA*), and strong promoters (*apm*, ermE*, *katE*, *moeE5*, *gapDH*).

Mutants carrying constructs with weak and medium strong promoters grew normally and reach the same final cell dry weight concentration as the empty vector control carrying pGUS and the wild-type of *Actinoplanes* sp. SE50/110, whereas mutants carrying strong promoters display growth deficiencies of different extent (Additional file 1: Data S3). This might be caused by side-effects due to the *gusA* overexpressing. In the case of the *actP* promoter from *S. coelicolor*, the mutant barely grew on maltose minimal medium, but displayed a strong signal in the GUS-assay (Additional file 1: Data S3). According to poor growth, RNA of sufficient yield and quality could not be isolated from this cultivation. Therefore, the strength of the *actP*-promoter could not be determined by RT-qPCR.

In conclusion, several promoters proved to be interesting candidates to be tested in pSET152 for the overexpression of *acb* genes in *Actinoplanes* sp. SE50/110. Additionally, we determined the transcription start sites of all promoters in the host background of *Actinoplanes* sp. SE50/110 by 5′-end specific transcriptome sequencing to identify the promoter motifs manually. These data are presented and discussed in Additional file 1: Data S4 (methods described in Additional file 1: Method S1 and Material S1).

### Transfer of promising promoters to the pSET152-expression system leads to the overexpression of the *acbC* gene

The *acbC* gene codes for a 2-*epi*-*5*-*epi*-valiolone synthase, which catalyzes the first step of acarbose biosynthesis, a cycling reaction forming 2-*epi*-5-epi-valiolone from *sedo*-heptulose-7P [7]. As this marks the transition from primary to secondary metabolism, overexpression of *acbC* gene has been expected to improve fluxes through the whole acarbose biosynthesis pathway. We transferred *acbC* into the pSET152-vector backbone, where it is expressed under control of medium or strong promoters obtained in the previous experiment (*rpsJ*, *efp*, *cgt*, *tipA*, *rpsL* and *gapDH*) or the native promoter of the first gene of the operon (*acbV*).

**Fig. 6:**
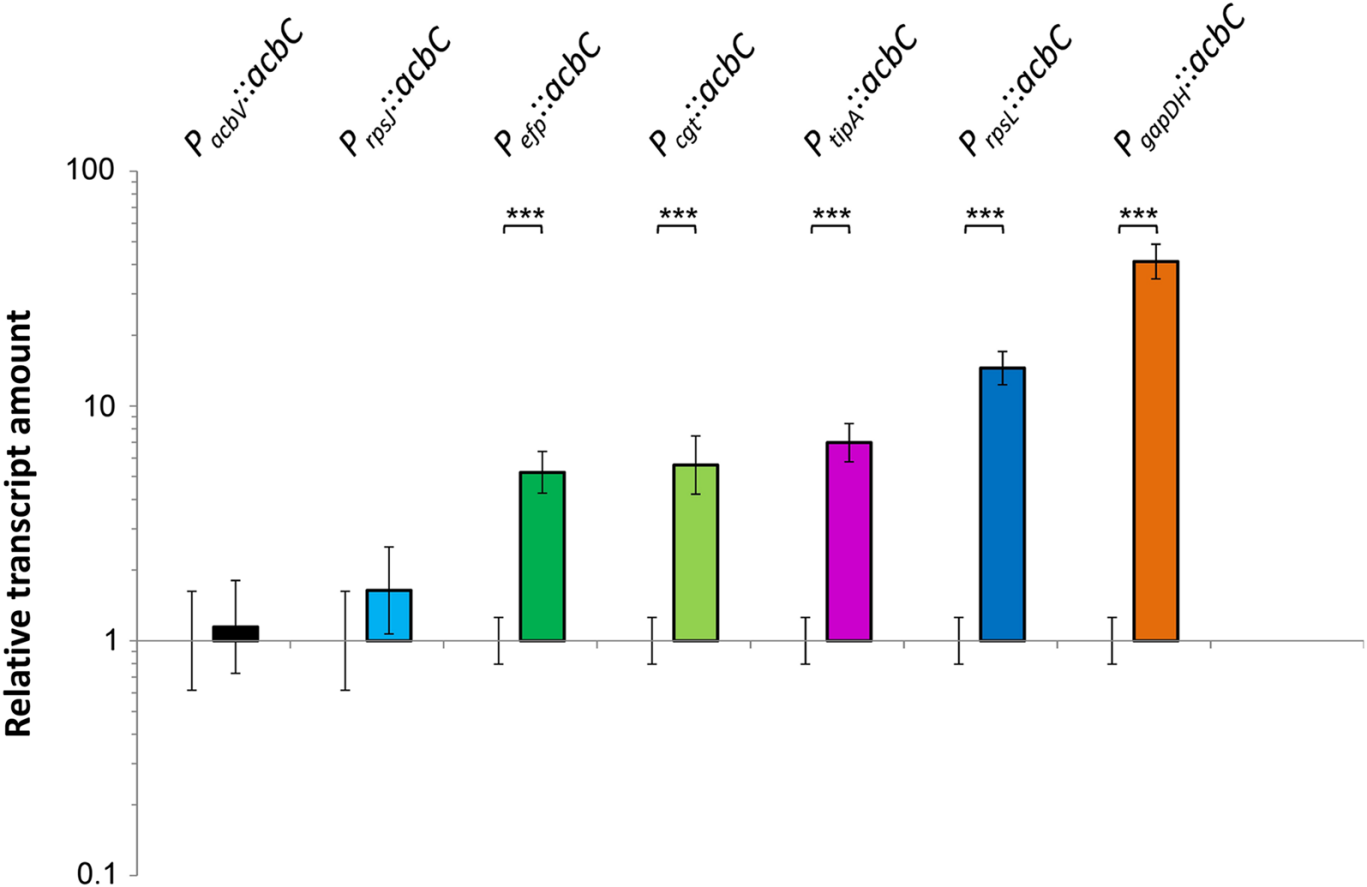
Relative transcript amounts of *acbC* gene under control of different homologous and heterologous promoters in *Actinoplanes* sp. SE50/110. The RNA was isolated from the growth phase of a shake flask cultivation in maltose minimal medium and analyzed by RT-qPCR. The transcript amounts were analyzed in relation to the empty vector control. Shown are the means and standard deviation of at least three biological replicates. The RT-qPCR indicates significant increase of gene expression compared to the empty vector control (set to a value of 1), which was tested by a two-sided t-test (p-values: P_*acbV*_: 0.9503, P_*rpsJ*_: 0.6084, P_*efp*_: 7.769e−05, P_*cgt*_: 0.004097, P_*tipA*_: 2.958e−06, P_*rpsL*_: 1.89e−06, P_*gapDH*_: 2.426e−08)

The expression of the *acbC* gene was measured on transcript level by RT-qPCR (Fig. 6). Integration of strong homologous and heterologous promoters into the pSET152 construct led to a significant stronger gene expression compared to a construct with native promoter (Fig. 6). Thus, overexpression of the *acbC* gene in varying amounts was successfully achieved in the host *Actinoplanes* sp. SE50/110.

### Overexpression of the *acbC* gene leads to accumulation of a phosphorylated intermediate of acarbose biosynthesis

**Fig. 7:**
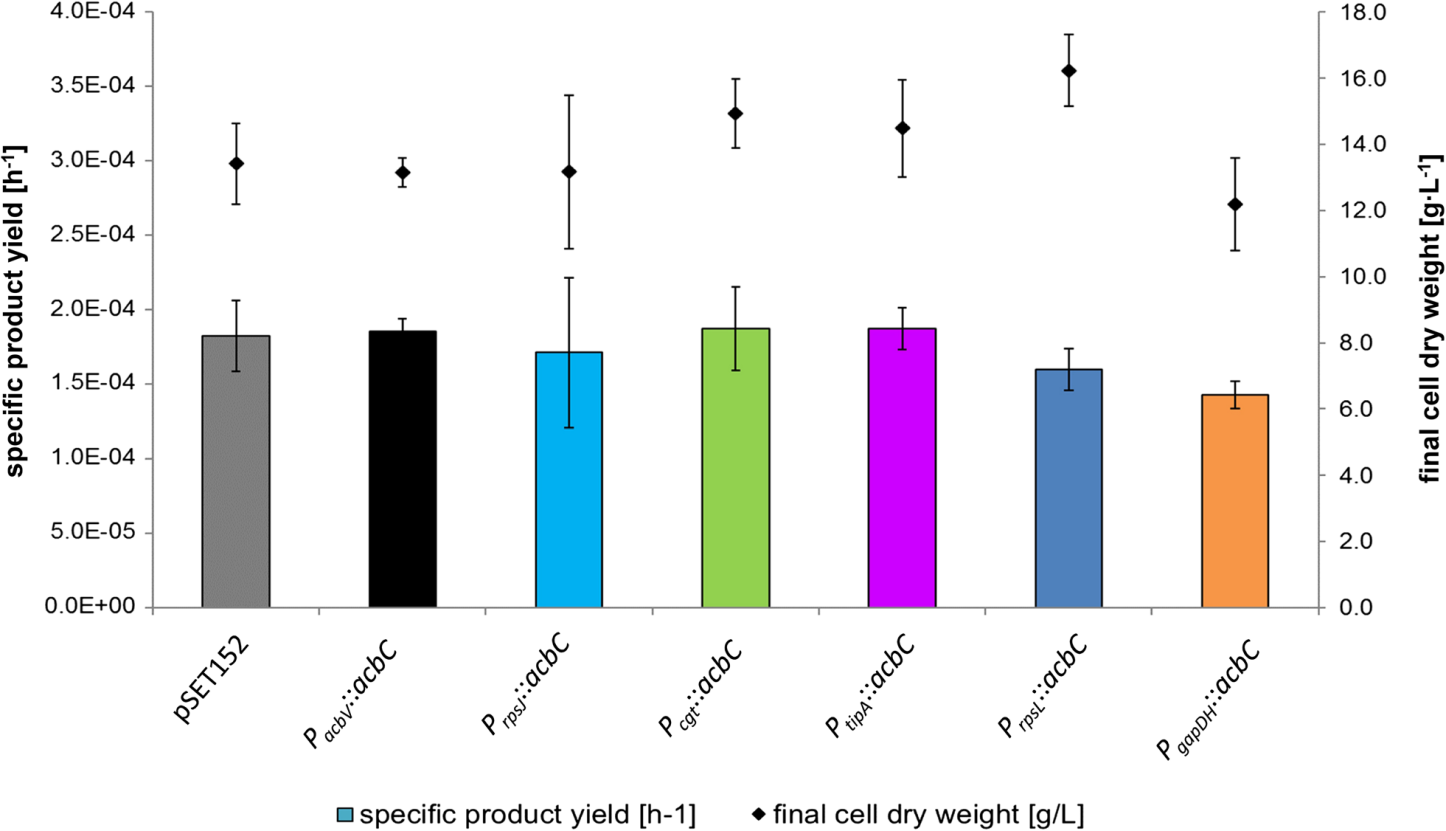
Shown are the specific product yields of pSET152-based *acbC*-overexpression mutants grown in a shake flask cultivation in maltose minimal medium in a bar diagram. Each specific product yield is calculated from the final acarbose concentration normalized to the final cell dry weight and cultivation time. Error was estimated by Gaussian error propagation. Final cell dry weights of each mutant are shown by a dot plot. Detailed growth curves and acarbose concentrations over time are shown in the Additional file 1: Data S5

The *acbC* overexpression mutants were analyzed in a shake flask cultivation in maltose minimal medium (Additional file 1: Data S5). Calculation of the specific product yields [h^−1^] shows, that overexpression of the *acbC* gene did not lead to an increase in acarbose production (Fig. 7). In the case of the strongest promoters P*rpsL* and P*gapDH*, specific product yields even tend to be reduced, which might give evidence, that high expression of AcbC could be detrimental for acarbose biosynthesis. However, these tendencies are not significant according to a two-sided t-test.

**Fig. 8:**
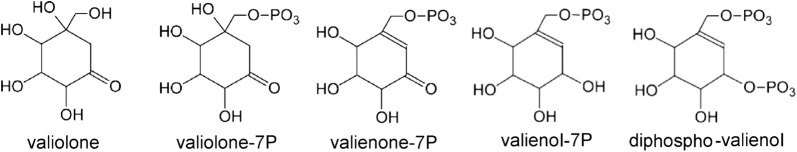
Connectivity of putative intermediates of bisphospho-valienol biosynthesis according to the model of acarbose biosynthesis developed by Zhang et al. [11]

**Fig. 9:**
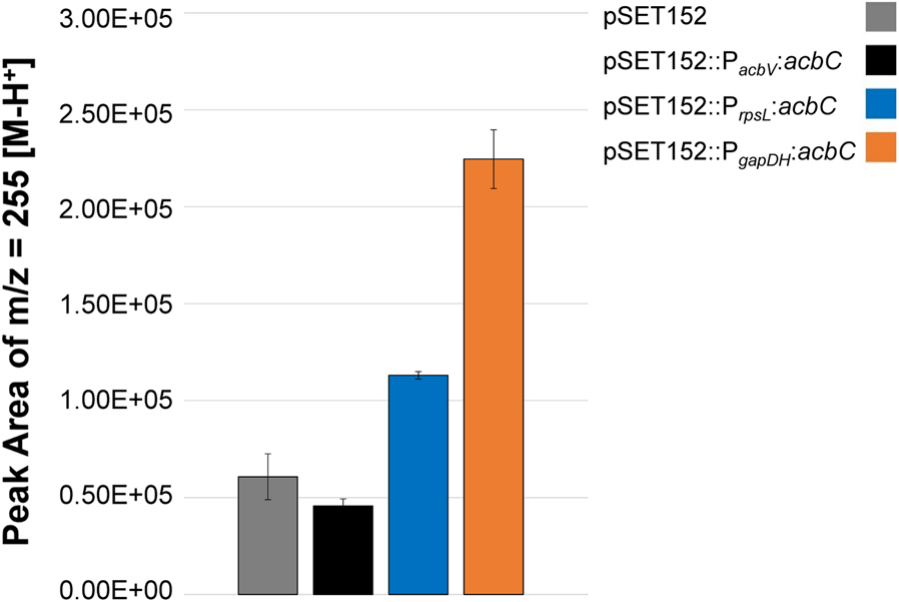
Peak areas of the mass m/z = 255.03 [M−H^+^] with the retention time 12.5 [min] of the LC–ESI–MS measurement in overexpression strains of the gene *acbC*. The compound is phosphorylated according to MS/MS-analysis (data not shown)

**Fig. 10:**
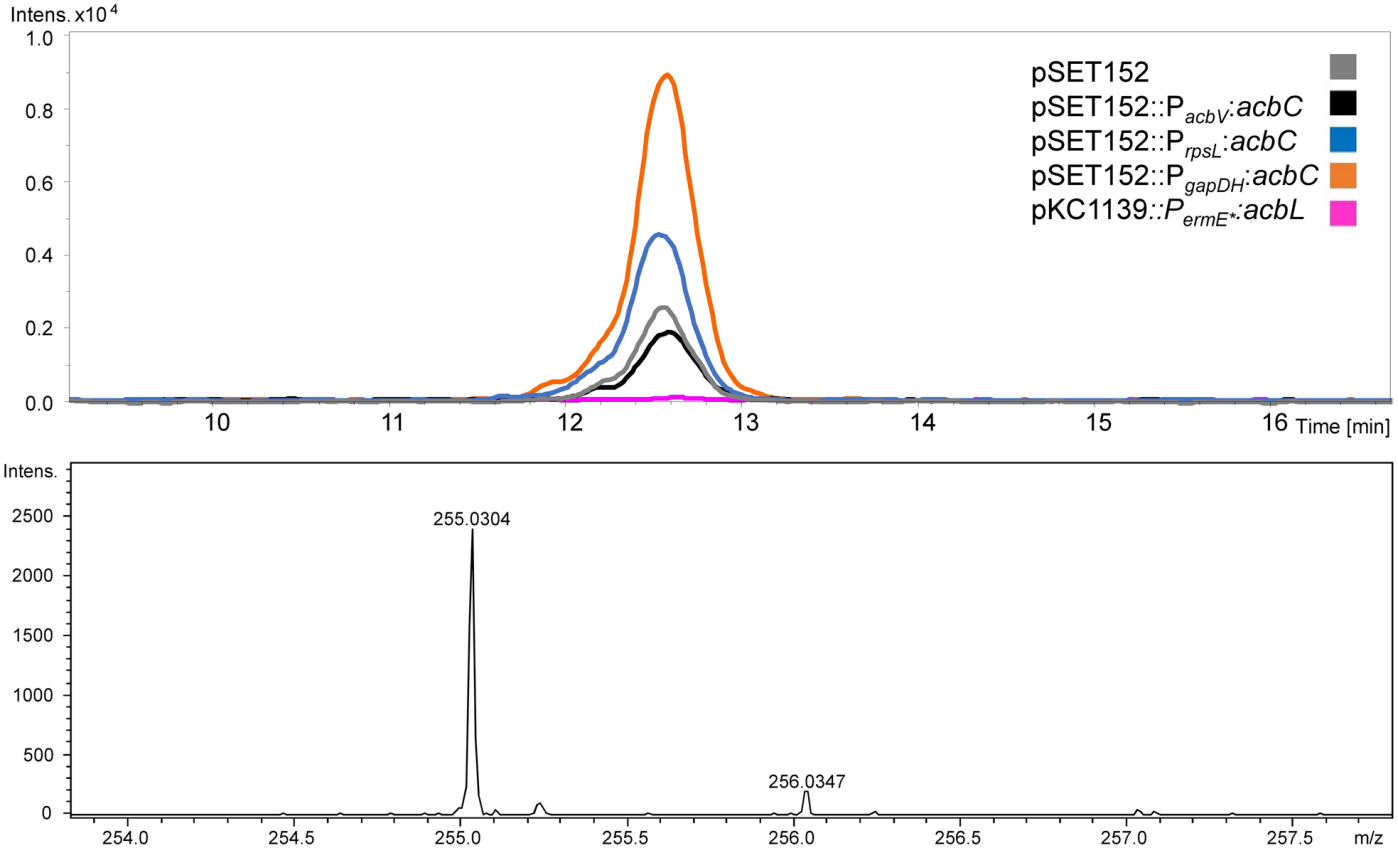
In the overexpression mutant of *acbC* (*gapDH*- or *rpsL*-promoter), a peak at 12.6 [min] arises (orange and blue), with the specific mass m/z = 255.03 [M−H^+^]. The corresponding peak is decisively higher compared to the empty vector control and a pSET152-construct with *acbC* under control of its native promoter (grey and black). According to MS/MS, this compound is phosphorylated (data not shown). In *Actinoplanes* sp. SE50/110 [pKC1139::P_ermE*_::*acbL*] (pink), in which the *acb* gene cluster was disrupted by unintended vector integration, this compound does not occur

We performed LC–ESI–MS (liquid chromatography–electron spray ionization–mass spectrometry) analysis of intracellular metabolites in order to find intermediates of the bisphospho-valienol biosynthesis (Fig. 8). We found a compound of the specific mass to charge ratio m/z = 255.03 [M−H^+^] accumulating in case of *acbC*-overexpression (Figs. 9 and 10). This accumulation corresponds to the promoter strength used for overexpression: the stronger is the promoter, the higher is the peak area of mass m/z = 255.03 [M−H^+^] (Fig. 9). The compound is phosphorylated according to tandem mass spectrometry (MS/MS) analysis (data not shown).

As this compound does not occur in the pKC1139-integration mutant, in which the *acb* gene cluster is disrupted by unintended vector integration (here analyzed for pKC1139::P_ermE*_::*acbL*, Fig. 10), we conclude, that compound m/z = 255.03 [M−H^+^] is part of the bisphospho-valienol biosynthesis. SmartFormula analysis (Bruker Daltonik GmbH, Bremen, Germany) gave the statistically most valid result for the formula C_7_H_12_O_8_P, which corresponds to valienol-7-phosphate (Additional file 1: Data S6).

Taken all evidence together, the identified mass m/z = 255.03 [M−H^+^] can be assigned to valienol-7P (1-*epi*-valienol-7P in the model Fig. 2).

In summary, no improved acarbose formation could be accomplished by *acbC* overexpression in *Actinoplanes* sp. SE50/110. Indeed, in case of strong overexpression, specific product yields even tend to be slightly reduced (Fig. 7).

On the one hand, this might be caused by an imbalance of acarbose biosynthesis enzymes. The synthesis of aminoglycosides is based on monofunctional enzymes catalyzing single steps [3]. Proteome analyses by Wendler et al. [9] have indicated, that the biosynthesis of acarbose and related metabolites takes place at the inner membrane of the cell, which is supporting the idea of enzymatic arrays modifying the metabolite and transferring it to the next enzyme step by step (substrate channeling). In the case of strong overexpression of single enzymes, it is likely, that the stoichiometry is disturbed leading to an imbalance of this enzymatic treadmill.

On the other hand, reduced acarbose formation might be as well affected by feedback inhibition by intermediates of the acarbose biosynthesis pathway, which potentially accumulate in an unbalanced system. Indeed, we found accumulation of a phosphorylated compound by LC–MS respectively MS/MS, which we strongly assume to be an intermediate of bisphospho-valienol-synthesis, as this compound vanishes in case of *acb* gene cluster disruption. The specific mass m/z = 255.03 [M−H^+^] was assigned to valienol-7P (corresponding to 1-*epi*-valienol-7P in the model Fig. 2). According to the recent acarbose biosynthesis model, this intermediate undergoes second phosphorylation by either the 1-epi-valienol-7P-kinase AcbU and/or the hydrolase AcbJ before being further nucleotidylated (unproven hypothesis, Wehmeier and Piepersberg [6]). In conclusion, accumulation of this compound in case of *acbC* overexpression might depict a bottleneck in the AcbU/J-mediated reaction during acarbose biosynthesis.

## Conclusion

The natural producer of acarbose, *Actinoplanes* sp. SE50/110, has been extensively studied in the last decades. As to date, no appropriate expression system for the overexpression of *acb* genes exists, here, we tested, evaluated and discussed different strategies.

The replicative pSG5-based vector pKC1139 turned out to be not suitable for expression of homologous genes in *Actinoplanes* sp. SE50/110, as unwanted vector integration by homologous recombination occurs and seems to be a favored process, putatively due to the high metabolic costs of vector replication.

However, the integrative φC31-based vector pSET152 is easy to handle and leads to fast, stable and effective transfer of genetic elements into the genome of *Actinoplanes* sp. SE50/110 [17]. As the acarbose biosynthesis genes are strongly transcribed and underlie strict regulation in the host [14, 32], simple gene duplication by pSET152-integration under control of native promoters has shown to be insufficient for overexpression of these genes. In a promoter screening experiment, the promoter strengths of 13 homologous and heterologous promoters were analyzed on protein level, of which 12 were analyzed on transcript level. Six promoters were selected to achieve overexpression of the gene *acbC*, the gene product of which catalyzes the first step of acarbose biosynthesis.

Surprisingly, by overexpression of the *acbC* gene, no enhanced specific product yields of acarbose were measured. This might indicate either disturbance of substrate channeling by oversupply of AcbC or a negative feed-back inhibition by one of the intermediates of bisphospho-valienol synthesis—presumably valienol-7P, which accumulates in the *acbC*-overexpression mutants. A co-overexpression of the genes *acbJ* and/or *acbU* might be considered for future improving acarbose biosynthesis, as these were proposed to catalyze the subsequent steps in acarbose biosynthesis, the second phosphorylation of 1-*epi*-valienol-7P to 1,7-diphospho-1-*epi*-valienol (compare to Fig. 2).

By knowledge of the strength of several promoters in the host *Actinoplanes* sp. SE50/110, another future strategy would be the integration of these promoters directly in front of the target genes by CRISPR/Cas9-technique. Such techniques have already been successfully tested for the integration of *acbC* gene into the strongly transcribed locus of *cgt* in *Actinoplanes* sp. SE50/110, displaying similar transcript amounts like achieved by pSET152-integration with *cgt*-promoter (unpublished). However, although suitable as integration tool, genetic engineering by CRISPR/Cas9 is cost- and time-consuming. For fast screening of overexpression mutants, f. e. for the detection of intermediate products, the pSET152-expression system is still the best tool, as it allows easy screening of several promoters in parallel.

## Methods

### Media and cultivation conditions of *Actinoplanes* sp. SE50/110

#### Preparation of glycerol stocks and spore solutions of *Actinoplanes* sp. SE50/110

For preparation of glycerol stocks, *Actinoplanes* sp. SE50/110 (ATCC 31044) was grown in the complex medium NBS (11 g L^−1^ glucose × 1H_2_O, 4 g L^−1^ peptone, 4 g L^−1^ yeast extract, 1 g L^−1^ MgSO_4_·7H_2_O, 2 g L^−1^ KH_2_PO_4_, 4 g L^−1^ K_2_HPO_4_) and mixed 2:3 with sterile 86% (v/v) glycerol. Glycerol stocks are stored at − 80 °C. A spore solution was prepared from solid culture, like described by Wolf et al. [18].

#### Preparation of minimal medium

Maltose minimal medium (72.06 g L^−1^ maltose·1H_2_O, 5 g L^−1^ (NH_4_)_2_SO_4_, 0.184 g L^−1^ FeCl_2_·4H_2_O, 5.7 g L^−1^ Na_3_C_6_H_5_O_7_·2H_2_O, 1 g L^−1^ MgCl_2_·6H_2_O, 2 g L^−1^ CaCl_2_·2H_2_O, trace elements (final concentration: 1 µM CuCl_2_, 50 µM ZnCl_2_, 7.5 µM MnCl_2_ dissolved in 1 M HCl) and phosphate buffer consisting of 5 g L^−1^ each K_2_HPO_4_ and KH_2_PO_4_ in aqua distilled) was prepared and filter sterilized following the protocol of Wendler et al. [13].

#### Shake flask cultivation

Cultivation was performed in 250 mL Corning^®^ Erlenmeyer baffled cell culture flasks at 28 °C and 140 rpm for 7 days. For inoculation of 50 mL medium, 1 mL spore solution of an OD = 3–5 was used. Cell dry weights were determined like described by Wolf et al. [32]. The supernatant was stored for later analysis at −20 °C.

### Acarbose quantification from the supernatant by high performance liquid chromatography measurement (HPLC)

The supernatant of maltose-grown cultures of *Actinoplanes* ssp. was centrifuged (20,000×*g*, 2 min), mixed 1:5 with methanol by vortexing and centrifuged again to remove precipitate (20,000×*g*, 2 min). The samples were transferred to HPLC vials and analyzed in the HPLC system 1100 series of Agilent (G1312A Binary Pump Serial#DE43616357, G1329A ALS autosampler Serial#DE43613/10, G1315A diode-array detector (DAD) Serial#DE72002469). As stationary phase the Hypersil APS-2 column (125 × 4 mm, 3 µm particle size) of Thermo Fisher Scientific Inc. (Waltham, Massachusetts, USA) was used, heated to 40 °C. As mobile phase an isocratic flow of 1 mL min^−1^ 68% acetonitrile (ACN) (solvent B) and 32% phosphate buffer (0.62 g L^−1^ KH_2_PO_4_ and 0.38 g L^−1^ Na_2_HPO_4_·2H_2_O) (solvent A) was applied. 40 µL of each sample was injected and separated in a 10 min run. Detection of acarbose was carried out with a DAD detector at 210 nm (reference 360 nm) and quantified from the peak areas of a calibration curve.

### Recombinant DNA work

Plasmid construction and assembly was performed by Gibson Assembly [53]. Fragments were amplified by PCR with the Phusion^®^ High-Fidelity PCR Master Mix with GC Buffer (NEB, Ipswich, MA, USA) and treated with *Dpn*I (Thermo Fisher Scientific, Waltham, MA, USA), when necessary. Purification of PCR products and gel extracts was performed by use of the NucleoSpin^®^ Gel and PCR Clean-up kit (Macherey-Nagel, Düren, Germany). The DNA fragments were mixed equimolar and added in a ratio of 1:4 to the Gibson Assembly Master Mix consisting of 0.64 µL T5 Exonuclease (10 U µL^−1^, NEB, Ipswich, MA, USA), 20 µL Phusion High-Fidelity DNA Polymerase (2 U µL^−1^, Thermo Fisher Scientific, US) and *Taq* DNA Ligase (NEB, Ipswich, MA, USA), 699.36 µL aqua distilled and 320 µL isothermal reaction buffer (25% PEG-8000, 1 mL 1 M Tris–HCl, 100 µL 1 M MgCl_2_, 100 µL 1 M DTT, 20 µL each 1 mM dNTP, 200 µL NAD). The sample was incubated at 50 °C for at least 1 h and subsequently transferred to *Escherichia coli* DH5αMCR by chemical transformation according to Beyer et al. [54]. Selection of *E. coli* was performed on Luria/Miller broth (LB)-media with 15 g L^−1^ agar–agar (Carl Roth, GmbH&Co.KG, Karlsruhe, Germany) and 50 mg L^−1^ apramycin-sulfate. Positive colonies were tested by PCR and gel-electrophoresis and by Sanger sequencing in-house sequencing core facility. Primers for control PCR and Sanger sequencing are listed in Additional file 1: Material S2.

### Construction of pKC1139 expression system

For construction of pKC1139-based expression plasmids [20], backbone amplification was performed by PCR by use of the primers: pKC1139EE_GAF (5′-CCCATGGCCATTCGAATTCGTAATC-3′) and pKC1139EE_GAR (5′-CGCTGGATCCTACCAACC-3′). As template, the modified backbone pKC1139EE from Julian Droste (unpublished) was used, which includes the promoter ermE* from *Saccharopolyspora erythraea* [51, 52]. This has shown to be active in *Actinoplanes* sp. SE50/110 [23].

Primers and templates for the inserts are listed in Additional file 1: Material S3. Genomic DNA of *Actinoplanes* sp. SE50/110 (ATCC 31044) was used as template DNA.

#### Construction of pSET152 expression system by use of native promoters

Backbone amplification was performed by use of the primers pSET152_GAF (5′-ATCCGCTCACAATTCCACAC-3′) and pSET152_GAR (5′-CCATCGGCGCAGCTATTTAC-3′). Primers for each insert are listed in Additional file 1: Material S4. Genomic DNA of *Actinoplanes* sp. SE50/110 (ATCC 31044) was used as template DNA. The genes *acbC* and *acbS* are part of the main operon of the *acb* gene cluster without own promoter. Therefore, the promoter of the gene *acbV* was used. For this, the construct pSET152::P_*acbV*_ was cloned and utilized as template for the amplification of a pSET152-backbone containing the *acbV*-promoter. For this, the primers pSET_PnatV_lin_GAF (5′-CGGAACCGCCGCCGGGTCGC-3′) and pSET_PnatV_lin_GAR (5′-ACAACATACGAGCCGGAAG-3′) were used.

#### Construction of plasmids for the gusA reporter system

For the promoter screening experiment, the pGUS-system of Myronovskyi et al. [43] was used, in which the reporter *gusA* was cloned into the vector pSET152. In pGUS, the reporter gene is not transcribed (no promoter). Three constructs were obtained from Dr. Liliya Horbal and Dr. Andriy Luzhetskyy from Saarland University (Saarbrücken, Germany): pSETGUS with the promoter of *tipA* from *S. lividans* [43], pSETPmoeE5 with the promoter of *moeE5* from *S. ghanaensis* [19], pSETPcdaRgusA with the promoter of *cdaR* from *S. coelicolor* [19]. One construct was cloned by use of the primer design of Horbal et al. [19] (pSETactPgusA with the promoter *actP* from *S. coelicolor*) by classical cloning with restriction of the vector backbone by *Xba*I and *Kpn*I.

All other promoter screening constructs were designed and cloned in this work by Gibson Assembly. Primers are listed in Additional file 1: Material S5. For linearization and amplification of the backbone, the primers pGUS_fwd (5′-AGCAACGGAGGTACGGACATGCTGCGGCCC-3′) and pGUS_rev (5′-CGACTAGTGCCAATAAGCTTGGTACCAATG-3′) were used.

#### Construction of pSET152 overexpression system with strong promoters

A total of 6 promoters were introduced into pSET152 in front of the gene *acbC* (*ACSP50_*3607). For this purpose, the construct pSET152::P_*acbV*_:*acbC* was used as backbone, in which the gene *acbC* is expressed under control of the native promoter of *acbV*. Both, backbone and insert, were amplified with primers containing overlaps to each other and annealed in a Gibson Assembly (Additional file 1: Material S6). Due to the small size of the promoter P*tipA* from *S. lividans*, the promoter sequence was attached to the primer sequence designed for the amplification of the vector backbone, and the vector pSET152::*PtipA*:*acbC* was obtained by amplification and self-annealing. For *rpsJ*-promoter a different design was used, because a template of pSET152 with the native promoter of *rpsJ* (*ACSP50*_0690) already existed (J. Droste, unpublished) (Additional file 1: Material S6).

### Conjugal transfer to *Actinoplanes* sp. SE50/110

Competent *Actinoplanes* sp. SE50/110 cells were prepared from freshly grown NBS-culture (see above). Cells were washed twice in 10% (w/v) ice-cold sucrose and twice in ice-cold 15% (v/v) glycerol. Finally, the cells were taken up in 15% (v/v) ice-cold glycerol (by addition of round about the fourfold volume of the pellet), aliquoted to 100 µL in reaction tubes and snap-frozen in liquid nitrogen. The competent *Actinoplanes* cells are stored at − 80 °C.

For conjugation, *Escherichia coli* ET12567/pUZ8002 [24] was used. After transfer of the desired construct into *E. coli* ET12567/pUZ8002 according to Beyer et al. [54] and selection on LB agar plates supplemented with 50 mg L^−1^ apramycin-sulfate, 50 mg L^−1^ kanamycin-sulfate and 15 mg L^−1^ chloramphenicol, cells were grown in liquid culture (LB-medium with the same supplements) and harvested at an optical density of 0.4–0.6. The cells were washed twice in ice-cold LB medium and mixed with competent cells of *Actinoplanes* sp. SE50/110. The cell suspension was plated on SFM agar plates. After 20–24 h of incubation at 28 °C, 1 mL 500 mg L^−1^ apramycin-sulfate dissolved in aqua distilled was distributed on the plate with a sterile swab. First exconjugants of *Actinoplanes* sp. SE50/110 can be observed after 1 week. Exconjugants were transferred to a SFM agar plate supplemented with 50 mg L^−1^ apramycin-sulfate. Re-streaking is performed for several times to purify *Actinoplanes* exconjugants from *E. coli.* To expedite this process, 50 mg L^−1^ fosfomycin or trimethoprim can be supplemented to the plate to get rid of the donor strain.

### Promoter screening experiment by spectrophotometric measurement of the glucuronidase activity

Two different types of glucuronidase assay were carried out: one with protein raw extract and one with entire cells. The protocol described here was adapted to *Actinoplanes* sp. SE50/110, according to protocols of Horbal et al. [19] and Siegl et al. [51]. The substrate 5-bromo-4-chloro-3-indolyl-β-d-glucuronide (X-Gluc) was chosen, as the substrate *p*-nitrophenyl-d-glucuronide turned out to dissociate under our assay conditions.

#### Growth conditions and sample preparation

*Actinoplanes* mutants carrying promoter-constructs with *gusA* gene, were cultivated for 1 week in maltose minimal medium, like described above. 500 µL of each culture was sampled for an assay with entire cells. 1 mL was sampled for an assay with protein raw extract and transferred to a screw cap tube containing zirconia/silica micro beads (Bio Spec Products Inc., Bartlesville, USA) of the sizes 0.1 mm and 0.05 mm. Cells were disrupted in a homogenizer (FastPrep FP120, Thermo Fisher Scientific, Waltham, MA, USA) for two times 30 s at speed setting 6.5 and 5 min on ice in between. After centrifugation, the lysate was transferred to a new reaction tube and centrifuged. The supernatant was used for a cell-free assay. Total protein quantification was carried by Bradford assay (Roti^®^-Nanoquant, Carl Roth GmbH&Co.KG, Karlsruhe, Germany). Samples and BSA standards were measured in a 200 µL reaction volume in a multititer plate (flat-bottom Nunc™ 96-Well Polystyrene Plates of Thermo Scientific, Waltham, MA, USA) in the Tecan reader Infinite M200 (Ref 30016056, Tecan Group AG, Männedorf, Schweiz) according to manufacturer’s protocol.

#### Glucuronidase (GUS) assay

The GUS assay was performed in a black microtiter plate (96 well PS F-bottom µCLEAR, black, med. binding, Greiner Bio-One, Kremsmünster, Österreich, REF 655096). 100 µL of each sample (either cell suspension or lysate) was pipetted in three wells, of which one serves as negative control and two as technical replicates. Gus-buffer (50 mM phosphate buffer pH 7.0 (5.136 g Na_2_HPO_4_·2H_2_O, 3.299 g NaH_2_PO_4_·2H_2_O) with 5 mM DTT and 0.1% Triton-X-100) was complemented with 2 mM substrate 5-bromo-4-chloro-3-indolyl-β-d-glucuronide (short: X-Gluc) (stock solution: 0.2 M in DMF), of which 100 µL was added to 100 µL of the sample. For the negative control, 100 µL Gus-buffer without substrate was added. Beside of the individual negative control of each sample, also medium and substrate controls was measured. The microtiter plate was measured in a pre-warmed Tecan reader Infinite M200 (Ref 30016056, Tecan Group AG, Männedorf, Switzerland) (37 °C) for 3 h (assay with entire cells), respectively for 2 h (assay with lysate). The absorption maxima of indigo were measured at 610 and 660 nm. After discounting the absorption value of each control and the substrate and medium controls, the slope of each absorption curve was calculated by linear regression and normalized either on cell dry weight (assay with entire cells) or on whole protein amount (assay with lysate). The normalized slope was used to compare β-glucuronidase activity.

### RNA isolation and reverse transcription quantitative PCR

#### Sampling and RNA isolation

For transcriptome analysis, 2 × 1 mL samples from *Actinoplanes* culture were taken during growth phase, separated from the supernatant by centrifugation (10 s) and snap-frozen in liquid nitrogen. Pellets were stored at − 80 °C until further processing.

For RNA isolation, frozen cell pellets were resuspended in 500 μL LB-buffer (NucleoSpin^®^ RNA Plus, Macherey–Nagel, Düren, Germany) and transferred to 2 mL lysing matrix tubes (0.1 mm spherical silica beads, MP Biomedicals, Santa Ana, California, USA). Cell disruption was carried out in a homogenizer (FastPrep FP120, Thermo Fisher Scientific, Waltham, MA, USA) for three times 20 s at speed setting 6.5 and 5 min on ice in between. Subsequently, the cell suspension was centrifuged for 5 min at 13,000×*g* and 4 °C. The supernatant was used for RNA extraction using the NucleoSpin^®^ RNA Plus Kit in combination with rDNase Set (Macherey-Nagel, Düren, Germany) for an on-column DNA digestion. After clean-up and elution according to manufacturer’s protocol, the DNA-digestion was repeated in-solution and the sample cleaned up again by use of the same kit. Residual DNA was tested negatively with two primer pairs binding to genomic DNA of *Actinoplanes* sp. SE50/110 and amplifying small fragments at round about 200–300 nt. The quantity of RNA was analyzed with the NanoDrop 1000 spectrometer (Peqlab, Erlangen, Germany).

#### Reverse transcription quantitative PCR

Reverse transcription quantitative PCR was carried out according to the protocol of Wolf et al. [32] by use of SensiFast SYBR No-Rox One-Step Kit (Bioline, London, UK) and 96 well lightcycler plates (Sarstedt, Nümbrecht, Germany) in a LightCycler 96 System of Roche (Mannheim, Germany). The relative RNA amount was normalized on total RNA (100 ng) and calculated as 2^−ΔCq^. ΔCq was calculated as the difference of the mean Cq in the mutant strain compared to the control strain. For determination of the relative transcription of a gene, the primers listed in Additional file 1: Material S7 were used.

### Liquid chromatography-mass spectrometry (LC–MS) measurements

#### Sample preparation

Triplicates of *Actinoplanes* sp. SE50/110 strains were grown in maltose minimal medium for 4 days. 10 mL of the culture were quickly filtrated through filtering paper by a Büchner funnel and water-jet pump and washed with 2.63 g L^−1^ NaCl solution. Cells were transferred into a pre-weighted round bottom screw-cap tubes, snap-frozen in liquid nitrogen and stored at − 80 °C. Cells were dried overnight in the Centrifugal Evaporator (SpeedVac) of Thermo Fisher Scientific (Waltham, MA, USA). 4 mg dried cells were transferred into a fresh 2 mL screw-cap tube and round about 500 µL of a mixture of zirconia/silica micro beads of the sizes 0.1 mm, 0.05 mm and 0.01 mm (Bio Spec Products Inc., Bartlesville, USA) were added. 700 µL 80% MeOH was added to the cells and beads. Cell disruption was carried out in a homogenizer (FastPrep FP120, Thermo Fisher Scientific, Waltham, MA, USA) for three times 30 s at speed setting 6.5. Samples were cooled for 5 min on ice in between. The cell suspension was centrifuged for 5 min at 13,000×*g* and 4 °C. 500 µL of the supernatant was transferred into HPLC vials, dried under nitrogen flow and taken up in 50 µL distilled water.

### LC–ESI–MS

For LC–MS, the LaChromUltra (Hitachi Europe Ltd., UK) HPLC system coupled to a microTOF-Q hybrid quadrupole/time-of-flight mass spectrometer (Bruker Daltonics, Bremen, Germany) was used, equipped with an electrospray ionization (ESI) source.

2 μL of the sample was separated with the SeQuant^®^ ZIC^®^-pHILIC 5 µm Polymeric column (150 × 2.1 mm) (Merck, Darmstadt, Germany). Eluent A (20 mM NH_4_HCO_3_, pH 9.3, adjusted with aqueous ammonia solution) and eluent B (acetonitrile) were applied at a flow rate of 0.2 mL min^−1^ by use of following gradient: 0 min B: 90%, 30 min B: 25%, 37.5 min B: 25%, 40.0 min B: 80%.

The ESI source was operated in negative ionization mode. The temperature of the dry gas and the capillary was set to 180 °C. The scan range of the MS was set to 200–1000 m/z.

## Additional file

**Additional file 1: Method S1.** 5′-library preparation, sequencing and data processing. **Material S1.** Adapters and primers used for 5′-library preparation. **Material S2.** Control primer for Colony PCR and Sanger sequencing. **Material S3.** Gibson Assembly primers for the amplification of inserts for pKC1139 expression system. **Material S4.** Gibson Assembly primer for the amplification of inserts with native promoters for pSET152. **Material S5.** Gibson Assembly primer for the *gusA* reporter system. **Material S6.** Gibson Assembly primer for *acbC* expression by strong promoters in pSET152. **Material S7.** Primers used in RT-qPCR. **Data S1.** PCR unveils vector-integration of pKC1139-constructs by homologous recombination. **Data S2.** Reduced transcription of *acb* genes downstream of the locus of vector integration shown by RT-qPCR for the mutant *Actinoplanes* sp. SE50/110 [pKC1139::P_ermE_*::*acbL*]. **Data S3.** Results of promoter screening by GUS-assay. **Data S4.** Determination of transcription start sites of heterologous promoters in *Actinoplanes* sp. SE50/110 by 5‘-end specific transcriptome sequencing. **Data S5.** Growth and acarbose formation of pSET152-based acbC-overexpression mutants. **Data S6.** Smart formula analysis of the isotopic pattern of mass = 255.03 [M−H^+^].

## Data Availability

All the data generated and analyzed in this study are included within the article (and its Additional file).
