# Supplementary material for: Evaluation of vector systems and promoters for overexpression of the acarbose biosynthesis gene acbC in Actinoplanes sp. SE50/110
Source: Microb Cell Fact. 2019 Jun 28;18:114. doi: 10.1186/s12934-019-1162-5 (PMC6599336; doi:10.1186/s12934-019-1162-5)
Supplement: Supplementary file 1 — Additional file 1: Method S1. 5′-library preparation, sequencing and data processing. Material S1. Adapters and primers used for 5′-library preparation. Material S2. Control primer for Colony PCR and Sanger sequencing. Material S3. Gibson Assembly primers for the amplification of inserts for pKC1139 expression system. Material S4. Gibson Assembly primer for the amplification of inserts with native promoters for pSET152. Material S5. Gibson Assembly primer for the gusA reporter system. Material S6. Gibson Assembly primer for acbC expression by strong promoters in pSET152. Material S7. Primers used in RT-qPCR. Data S1. PCR unveils vector-integration of pKC1139-constructs by homologous recombination. Data S2. Reduced transcription of acb genes downstream of the locus of vector integration shown by RT-qPCR for the mutant Actinoplanes sp. SE50/110 [pKC1139::PermE*::acbL]. Data S3. Results of promoter screening by GUS-assay. Data S4. Determination of transcription start sites of heterologous promoters in Actinoplanes sp. SE50/110 by 5‘-end specific transcriptome sequencing. Data S5. Growth and acarbose formation of pSET152-based acbC-overexpression mutants. Data S6. Smart formula analysis of the isotopic pattern of mass = 255.03 [M−H+]. [file 12934_2019_1162_MOESM1_ESM.docx]

# Additional Method

### **Method S1.** 5’-library preparation, sequencing and data processing

RNA of triplicates of each mutant were isolated from exponentially grown cultures and equimolar pooled (total amount of 10 µg RNA in 26 µL RNAse-free water). Stable RNA was depleted by use of the Ribo-Zero rRNA Removal Kit for bacteria (Illumina, San Diego, USA). The sample was analyzed by an Agilent RNA 6000 Pico chip in the Bioanalyzer (Agilent, Böblingen, Germany).

For fragmentation, 80 µL sample was treated with 20 µL fragmentation buffer (100 mM KOAc, 30 mM MgOAc in 200 mM Tris-HCl pH 8.1) and incubated for 3.5 min at 94 °C. For termination, 100 µL ice-cold fragmentation stop buffer (10 mM Tris, 1 mM EDTA, pH 8) was added and incubated on ice for 5 min. The fragmented RNA was cleaned up and concentrated by use of the RNeasy MinElute Cleanup Kit (Qiagen, Hilden, Deutschland), eluted in 18.5 µL RNAse-free water and analyzed in the Bioanalyzer (see above).

5’-library preparation was carried out according to the protocol of Pfeifer-Sancar *et al.* (2013) [55]. First, RNA was processed by a terminator exonuclease XRN-1 (NEB, Ipswich, MA, USA) to digest mono- and diphosphorylated transcripts leaving native transcripts with 5′ triphosphate end (5′-PPP). Reaction was carried out for 60 min at 37 °C and heat-inactivated for 10 min at 70 °C, according to the manufacturer’s instructions. The library was cleaned up and concentrated by use of RNeasy MinElute Cleanup Kit (Qiagen, Hilden, Deutschland). Second, the 5′-PPP-transcripts were converted to monophosphorylated transcripts (5’-P) by a RNA 5’-Polyphosphatase (Epicentre, Madison, USA). The reaction was performed according to manufacturer’s protocol for 45 min at 37 °C. The processed RNA-pool was precipitated. For this, the volume was adjusted to 180 μL using RNase-free water. 20 µL of 3 M sodium acetate (NaOAc, pH 5.2) and 2 µL glycogen (10 mg mL^-1^) were added and mixed by gentle vortexing. Precipitation was accomplished by addition of 600 µL ice-cold 100 % ethanol. The sample was inverted several times and stored overnight at -20 °C. The precipitate was centrifuged (>16,000 g at 4 °C for 30 min) and washed twice with 500 µL ice-cold 70 % (v/v) ethanol. The pellet was air-dried and taken up in 13 µL RNAse-free water. Next, adapters were ligated to the 5’-ends of the prepared RNA fragments by addition of 60 µM of RNA adapter (**Material S1**) and T4 RNA ligase 1 (NEB, Ipswich, MA, USA) according to manufacturer’s protocol. The library was cleaned up and concentrated by use of the RNeasy MinElute Cleanup Kit (Qiagen, Hilden, Deutschland). For reverse transcription and tagging of the 3’-end of the cDNA, a stem-loop DNA adapter was added into the reverse transcription reaction (**Material S1**). In advance, the loop primer was denatured for 3 min at 98 °C and cooled to 25 °C at a rate of 1 °C per 10 sec in a Mastercycler pro S (Eppendorf, Hamburg, Germany), like described by Pfeifer-Sancar *et al.* (2013) [55]. For reverse transcription, SuperScript^TM^ III First-Strand Reverse Transcriptase (Invitrogen, Carlsbad, USA) was used according to manufacturer’s instructions under addition of 5 µM of the prepared loop primer and 1 µL RNaseOUT™ Recombinant Ribonuclease Inhibitor (Invitrogen, Carlsbad, USA). Reaction was carried out for 30 min at 16 °C, 60 min at 50 °C and inactivated at 85 °C for 5 min. After cDNA synthesis, RNA was digested by RNase H (NEB, Ipswich, MA, USA) for 20 min at 37 °C. The cDNA library was amplified directly by use of the Phusion® High-Fidelity PCR Master Mix with GC Buffer (NEB, Ipswich, MA, USA). Primers for indexing are listed in **Material S1**. The library was cleaned up from a 1.5 % agarose gel (Certified™ Low Range Ultra Agarose, Bio-Rad, Hercules, USA) cut between 150–1,000 bp and purified by the QIAquick Gel Extraction Kit (Qiagen, Hilden, Deutschland). The library was eluted in 20 µL water and quantified by a DNA High Sensitivity Assay chip in the Bioanalyzer (Agilent, Böblingen, Germany).

The 5’-library was sequenced on a 2 x 75 nt MiSeq run (Illumina). Sequencing yielded about 14 million read pairs, which were quality-trimmed using Trimmomatic v0.3.5 [56]. Forward reads were mapped to a separate reference sequence for each integration mutant using Bowtie2 in single-end mode [57]. ReadXplorer was used for visualization and identification of transcription start sites [58].

# Additional Material

**Material S1.** Adapters and primers used for 5’-library preparation.

| **Name/function** |  | **sequence (5’-3’)** |
| --- | --- | --- |
| Ligation-5‘-Adapter 1 | RNA | CCCUACACGACGCUCUUCCGAUCGAG |
| Loop primer | DNA | AGATCGGAAGAGAGACGTGTGCTCTTCCGATCTNNNNNNN |
| PCR Primer 1 | DNA | AATGATACGGCGACCACCGAGATCTACACTCTTTCCCTACACGACGCTCTTCCGATCGAG |
| PCR Primer 2.1 | DNA | CAAGCAGAAGACGGCATACGAGAT**cgtgat**GTGACTGGAGTTCAGACGTGTGCTCTTCCGATCT |

**Material S2.** Control primer for Colony PCR and Sanger sequencing.

| **Primer name** | **Primer length** | **Primer sequence (5’-3’)** |
| --- | --- | --- |
| **Binding on the vector backbone** | | |
| pKC1139EE_seq1 | 18 | CCGGTTGGTAGGATCCAG |
| pKC1139EE_seq2 | 18 | ATGCTTCCGGCTCGTATG |
| pGUS_seq1 | 18 | AAGGATCGGGCCTTGATG |
| pGUS_seq2 | 19 | GACGGGCCGCAGCATGTCC |
| pSET_seq1 | 18 | GTCCTGCGGGTAAATAGC |
| pSET_seq2 | 20 | ACTGGAAAGCGGGCAGTGAG |
| pSET_seq2n | 20 | CAGCGTGAGCTATGAGAAAG |
| **Binding within the insert** | | |
| seq1_acbC | 18 | TGGCCGTTGAAGTTGACC |
| seq2_acbC | 18 | TCATCACCGCGAAGATCC |
| seq3_acbC | 19 | GATGGACGTGGCCGGTCTG |
| seq1_acbL | 18 | CGGTCTCCGGTGGCTTGG |
| seq1_acbM | 18 | GCCATCATCCGGGTGGTC |
| seq1_acbN | 18 | CTGCTTCGCCGCGGTCTC |
| seq1_acbQ | 19 | GCCGTCCTGCGTGGTGGTG |
| seq2_acbQ | 18 | AATCCGATGCACGCCTAC |
| seq3_acbQ | 18 | GTCGACCGGATCGATTTC |
| seq4_acbQ | 18 | ATCATCGGCGAGGATCTG |
| seq1_acbR | 18 | GACGCGCTGATCCGCAAG |
| seq2_acbR | 18 | GTCTCGTTCGTGGAGAAG |
| seq1_acbS | 18 | TCATCAGGTCGCACTTCG |
| seq2_acbS | 18 | TGCGCCGTCGGTGACGAG |
| seq3_acbS | 19 | GGGATGGCGCACTTCGGTC |
| seq1_WXY | 18 | CCTGCGAACCATGTTCTC |
| seq2_WXY | 18 | CTCGGCGCCCTCGGCAAC |
| seq3_WXY | 18 | ATCATCACCGAAGGACTG |
| seq4_WXY | 18 | AACCGGATCGACGAGATG |
| seq5_WXY | 18 | ATCAAGAACGCGCTCGTC |
| zwf1_seq1rev | 18 | ACGGCACGCCGGCCCAGC |
| zwf1_seq2rev | 18 | TAGTGGTCGATCCGGTAG |

**Material S3.** Gibson Assembly primers for the amplification of inserts for pKC1139 expression system.

| **insert** | **primer name** | **primer sequence (5’-3’)** | **product (bp)** |
| --- | --- | --- | --- |
| *acbR* (*ACSP50*_3597) | R_GAF | ggttggtaggatccagcgATGAGCACGGGCGTACG | 1130 |
|  | R_GAR | cgaattcgaatggccatgggTCATCGCCGGGCTCCGGTG |  |
| *acbQ* (*ACSP50*_3601) | Q_GAF | ggttggtaggatccagcgATGACCACCACGACGGATG | 2143 |
|  | Q_GAR | cgaattcgaatggccatgggCAGCGAGGTCAGGGTGTG |  |
| *acbK* (*ACSP50*_3602) | K_GAF | ggttggtaggatccagcgATGTCGGAGCACACCGACG | 958 |
|  | K_GAR | cgaattcgaatggccatgggCGGGTGGTGCGGTGGCCGCTTC |  |
| *acbM* (*ACSP50*_3603) | M_GAF | ggttggtaggatccagcgATGAAGCGGCCACCGCACCACCC | 1118 |
|  | M_GAR | cgaattcgaatggccatgggTCATCGCCCGACCAACGCTTC |  |
| *acbL* (*ACSP50_*3604) | L_GAF | ggttggtaggatccagcgATGAGCCGGCACCGCGCGATC | 1154 |
|  | L_GAR | cgaattcgaatggccatgggCCACCAGAGTCCCGCTCATC |  |
| *acbN* (*ACSP50*_3605) | N_GAF | ggttggtaggatccagcgATGAGCGGGACTCTGGTG | 807 |
|  | N_GAR | cgaattcgaatggccatgggCCCACCCGGCAGGTCACG |  |
| *acbO* (*ACSP50*_3606) | O_GAF | ggttggtaggatccagcgATGACCTGCCGGGTGGGGCTGAC | 864 |
|  | O_GAR | tcgaatggccatgggCTACCGTCTCGACACCACTC |  |
| *acbC* (*ACSP50*_3607) | C_GAF | ggttggtaggatccagcgATGAGTGGTGTCGAGACGGTAGG | 1253 |
|  | C_GAR | cgaattcgaatggccatgggCGGCGTCCGCGGCCCGAGCTAGG |  |

**Material S4.** Gibson Assembly primer for the amplification of inserts with native promoters for pSET152.

| **insert** | **primer name** | **primer sequence (5’-3’)** | **product (bp)** |
| --- | --- | --- | --- |
| *acbA* (*ACSP50*_3609) | pSETNat_A_GAFn | gtaaatagctgcgccgatggCCAATGGGTGCCCGATGTTC | 1045 |
|  | pSETNat_A_GARn | gtgtggaattgtgagcggatCGCCGCCCGGGCCGGTCACC |  |
| *acbB* (*ACSP50*_3608) | pSETNat_B_GAF | gtaaatagctgcgccgatggACCGACCATATCAGCAAG | 1088 |
|  | pSETNat_B_GAR | gtgtggaattgtgagcggatTTGCCGTCAGGTCCACCAGGAAC |  |
| *acbC* (*ACSP50*_3607) | pSET_acbC_Pvnat_GAF | gcgacccggcggcggttccgATGAGTGGTGTCGAGACGGTAGG | 1254 |
|  | pSET_acbC_Pvnat_GAR | cttccggctcgtatgttgtCGGCGTCCGCGGCCCGAGCTAGG |  |
| *acbS* (*ACSP50*_3596) | pSET_acbS_Pvnat_GAF | gcgacccggcggcggttccgATGCACATCATCGAGACGTACTTC | 2169 |
|  | pSET_acbS_Pvnat_GAR | cttccggctcgtatgttgtTCATGCCGTCACCTCGTC |  |
| *acbV* (*ACSP50_3594*) | pSETNat_V_GAF | gtaaatagctgcgccgatggCGATGCAAGAACTTGCTGAAAC | 1606 |
|  | pSETNat_V_GAR | gtgtggaattgtgagcggatTGTCATGCCGTCACCCGCCCGGCCTC |  |
| *acbWXY* (*ACSP50*_3591-3) | pSETNat_WXY_GAF | gtaaatagctgcgccgatggTCGCGGTCACATTTCGAGG | 3099 |
|  | pSETNat_WXY_GAR | gtgtggaattgtgagcggatTCAGCTGCCGGGCATCTCGTAG |  |
| cgt (*ACSP50*_5024) | cgt_GAF | gtaaatagctgcgccgatggCCTGACGGGTTCTGCACCTC | 881 |
|  | cgt_GAR | tgttgtgtggaattgtgagcggatGGATCAGTACGCGCCGAAGG |  |
| zwf1 (*ACSP50*_1790) | pSET_Pnat_zwf1_GAF | gtaaatagctgcgccgatggCGGCCTGTCGCGGAACACTC | 1629 |
|  | pSET_Pnat_zwf1_GAR | gtgtggaattgtgagcggatAGCCCGATCATGCTCGCCTC |  |

**Material S5.** Gibson Assembly primer for the *gusA* reporter system.

| **promoter of** | **template** | **primer name** | **primer sequence (5’-3’)** |
| --- | --- | --- | --- |
| cgt (ACSP50_5024) | ATCC 31044 | cgt_fwd | cattggtaccaagcttattggcactagtcgGCCCGGCCCTGTCGAGCTGA |
|  |  | cgt_rev | gggccgcagcatgtccgtacctccgttgctGACAGTCCCCTTTGATGATC |
| efp  (ACSP50_ 6465) | ATCC 31044 | efp_fwd | cattggtaccaagcttattggcactagtcgTGGAGCACATCTGCCGGTAG |
|  |  | efp_rev | gggccgcagcatgtccgtacctccgttgctAGGTCGTTGGTGGAAGCCAT |
| ACSP50_7457 | ATCC 31044 | 7457_fwd | cattggtaccaagcttattggcactagtcgGGGCGACACTCCGAAGGTGA |
|  |  | 7457_rev | gggccgcagcatgtccgtacctccgttgctGGAGGTTCTTGGCCGGTCAT |
| katE (ACSP50_3066) | ATCC 31044 | katE_fwd | cattggtaccaagcttattggcactagtcgATCTCGGGCTCGGTAGGCAT |
|  |  | katE_rev | gggccgcagcatgtccgtacctccgttgctCCGGACAAACTCCTCGATAA |
| rpsJ (ACSP50_0690) | ATCC 31044 | rpsJ_fwd | cattggtaccaagcttattggcactagtcgGTGGTGTTGCAGACTTCTTGAG |
|  |  | rpsJ_rev | gggccgcagcatgtccgtacctccgttgctCGGGTTTCTCCGCTCCCTTC |
| apm (aac(3)IV) | pCRISPomyces-2 [45] | aac(3)IV_fwd | cattggtaccaagcttattggcactagtcgCTCTGCTGAAGCCAGTTACC |
|  |  | aac(3)IV_rev | gggccgcagcatgtccgtacctccgttgctCAGTCGATCATAGCACGATCAAC |
| gapDH | pCRISPomyces-2 [45] | gapDH_fwd | cattggtaccaagcttattggcactagtcgGCTGCTCCTTCGGTCGGACGTGCGTCTAC |
|  |  | gapDH_rev | gggccgcagcatgtccgtacctccgttgctCTGAGAAGACTTGCGTATCCC |
| rpsL | pCRISPomyces-2 [45] | rpsL_fwd | cattggtaccaagcttattggcactagtcgTGAGCACGTCCGCGAGCTGG |
|  |  | rpsL_rev | gggccgcagcatgtccgtacctccgttgctTACGTCTCCGTCGTCTACTC |

**Material S6.** Gibson Assembly primer for *acbC* expression by strong promoters in pSET152.

| **fragment** | **template** | **size (bp)** | **primer sequence (5’-3’)** |
| --- | --- | --- | --- |
| pSETC_*cgt*P_lin | pSET152::Pn*V*: *acbC* (this work) | 6567 | ggatcatcaaaggggactgtcATGAGTGGTGTCGAGACGGTAG |
|  |  |  | caatcggctgctgatgacacgccCCATCGGCGCAGCTATTTAC |
| *cgt*P | ATCC 31044 | 253 | gtaaatagctgcgccgatggGGCGTGTCATCAGCAGCCGATTG |
|  |  |  | ctaccgtctcgacaccactcatGACAGTCCCCTTTGATGATCC |
| pSETC_*rpsL*P_lin | pSET152::Pn*V*: *acbC* (this work) | 6564 | gagtagacgacggagacgtaATGAGTGGTGTCGAGACGGTAG |
|  |  |  | cctgacttccgcctgcagggcCCATCGGCGCAGCTATTTAC |
| *rpsLP* | pCRISPomyces-2 [45] | 344 | gtaaatagctgcgccgatggGCCCTGCAGGCGGAAGTCAGG |
|  |  |  | ctaccgtctcgacaccactcatTACGTCTCCGTCGTCTACTC |
| pSETC_*efp*P_lin | pSET152::Pn*V*: *acbC* (this work) | 6564 | gagtcaagatcaaggcaggacATGAGTGGTGTCGAGACGGTAG |
|  |  |  | gctggtgagggcgaatcgggCCATCGGCGCAGCTATTTAC |
| *efp*P | ATCC 31044 | 116 | gtaaatagctgcgccgatggCCCGATTCGCCCTCACCAGC |
|  |  |  | ctaccgtctcgacaccactcatGTCCTGCCTTGATCTTGACTC |
| pSETC_*gapDH*P_lin | pSET152::Pn*V*: *acbC* (this work) | 6565 | gagtatctgaaaggggatacgcATGAGTGGTGTCGAGACGGTAGG |
|  |  |  | ccaaaaggagcctttaattgCCATCGGCGCAGCTATTTAC |
| *gapDH*P | pCRISPomyces-2 [45] | 358 | gtaaatagctgcgccgatggCAATTAAAGGCTCCTTTTGG |
|  |  |  | ctaccgtctcgacaccactcatGCGTATCCCCTTTCAGATACTC |
| pSET_*rpsJ*P_lin | pSET152_P*rpsJ* (J. Droste) | 5713 | ATCCGCTCACAATTCCACAC |
|  |  |  | GGTGGCTTCTGTTTCCTTCTC |
| acbC_*rpsJ*P | gDNA | 1253 | agaaggaaacagaagccaccATGAGTGGTGTCGAGACGGTAGG |
|  |  |  | cttccggctcgtatgttgtCGGCGTCCGCGGCCCGAGCTAGG |
| pSETC_*tipA*_lin | pSET152::Pn*V*: *acbC* (this work) | 6646 | GAGGCAGCGTGGACGGCGTGGTACCAAGCTTATTGGCACTAGTCGAGCAACGGAGGTATTCCGATGAGTGGTGTCGAGAC |
|  |  |  | CACGCCGTCCACGCTGCCTCCTCACGTGACGTGAGGTGCAAGCCCGGACGTTCTAGGGATCCATCGGCGCAGCTATTTAC |

**Material S7.** Primers used in RT-qPCR.

| **genetic locus** | **fwd-primer (5’-3’)** | **rev-primer (5’-3’)** | **fragment size (bp)** |
| --- | --- | --- | --- |
| *acbA* (*ACSP50*_3609) | TCATGCTCGGCGACAACCTG | GACCGGTTTCTCCTCGATGG | 173 |
| *acbB* (*ACSP50*_3608) | CCCGCTGCTCGAACAACTAC | CCGCCGATGTGATAGACCTC | 205 |
| *acbC* (*ACSP50*_3607) | GATCGCGCTGATCAAGGATG | CTGAACGTGTGCCCGTAGTC | 213 |
| *acbS* (*ACSP50*_3596) | GTTGCCGGACCGGTTCTATC | CCCGGTACACCGACTTGTTG | 248 |
| *acbW*(*ACSP50*_3593) | GGTGTACGACCGGAACATGC | GTTCGGCGTGGATGTGGTTG | 224 |
| *acbX (ACSP50_3592)* | TCGGGATGCTGCACACCAAC | CGACGCGAACATCGCGAAAC | 191 |
| *acbY* (*ACSP50*_3591) | TCCGAACGGTTCCTCTATCC | AACTCGCTGAGCTGGTTGAC | 239 |
| *cgt* (*ACSP50*_5024) | CACCACGTACTGGAACTC | GCGACCTTCAACGTGAC | 192 |
| *zwf1* (*ACSP50*_1790) | ACGCCGACTTCGACAAACTC | TCGTTGCCGAACGGCTTCTC | 202 |
| *gusA* | ACGCGGACATCCGCAACTAC | CCCTGGTGCTCCATCACTTC | 157 |

# Additional Data

## Data S1. PCR unveils vector-integration of pKC1139-constructs by homologous recombination.

**Data S1.1.** Expected PCR-fragment sizes for pKC1139-constructs containing different genes of interests by use of the primer combinations AB, CD, CB and AD (compare to **Figure 3**).

*
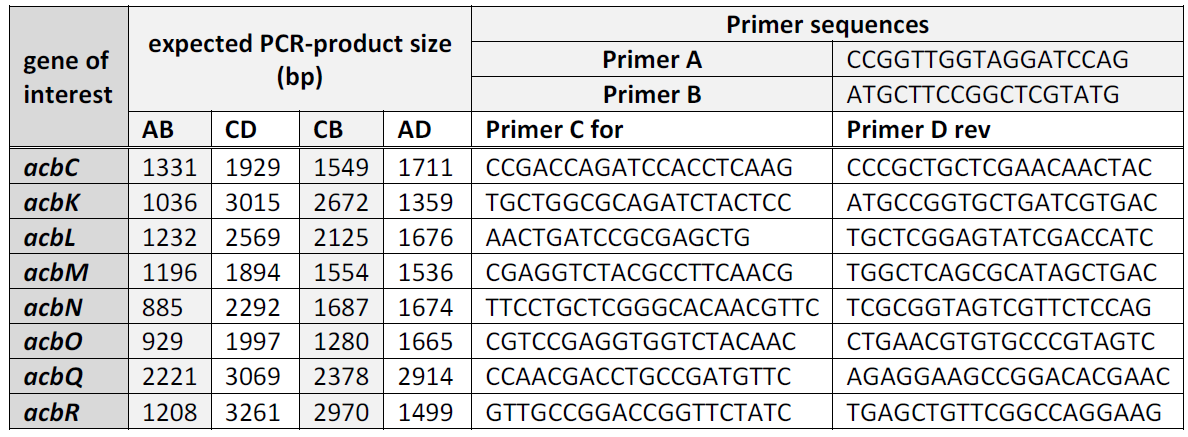
*


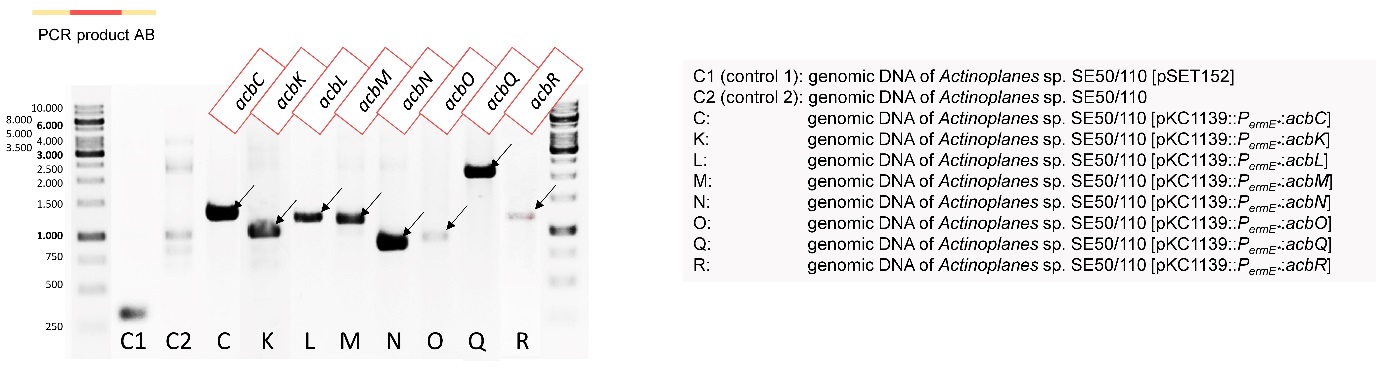


**Data S1.2.** Result of test-PCR shown for PCR-primer A and B displaying presence of a replicative vector containing the corresponding gene of interest in all cases.


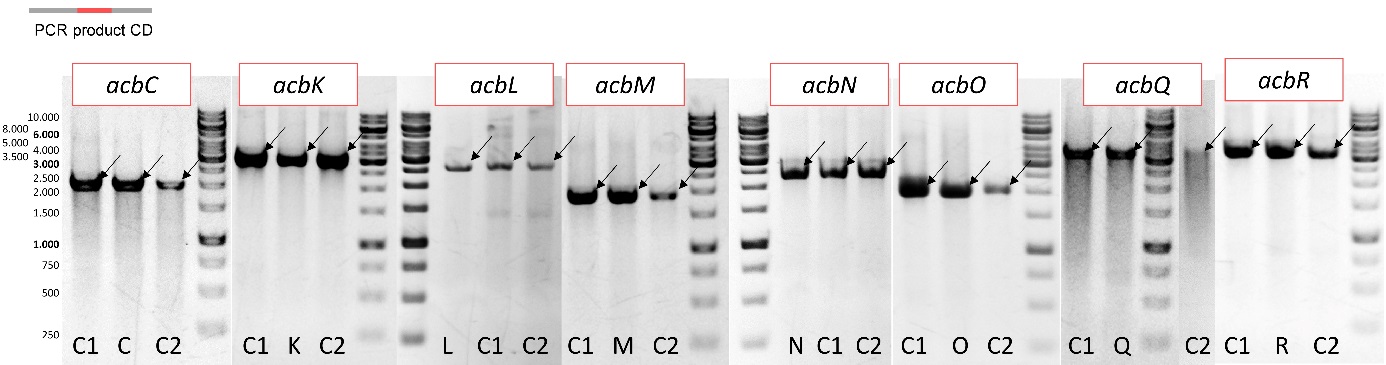


**Data S1.3.** Result of test-PCR shown for PCR-primer C and D displaying presence of cells with intact genetic locus of the gene of interest (without vector integration).


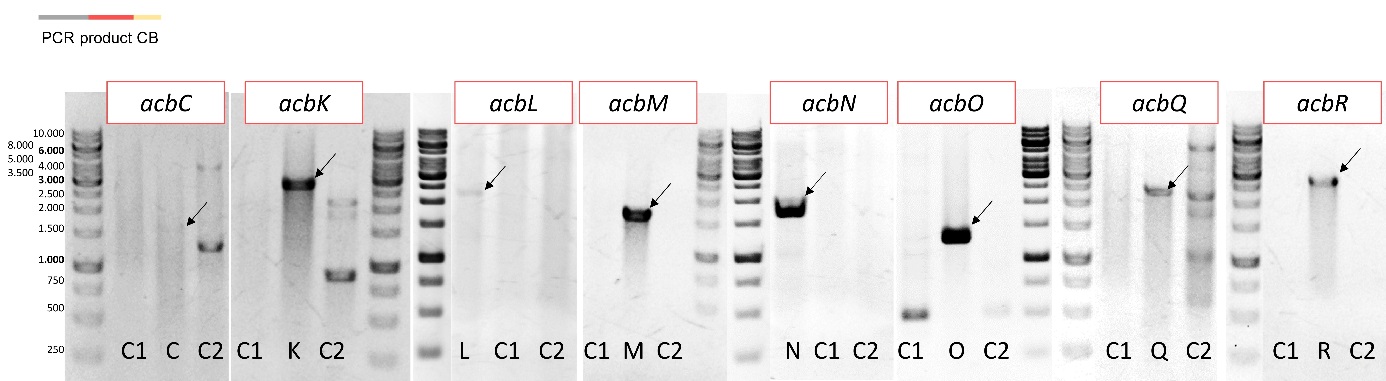


**Data S1.4.** Result of test-PCR shown for PCR-primer C and B displaying presence of cells with pKC1139-construct integrated into the genetic locus of the gene of interest (proof of vector integration in all cases).


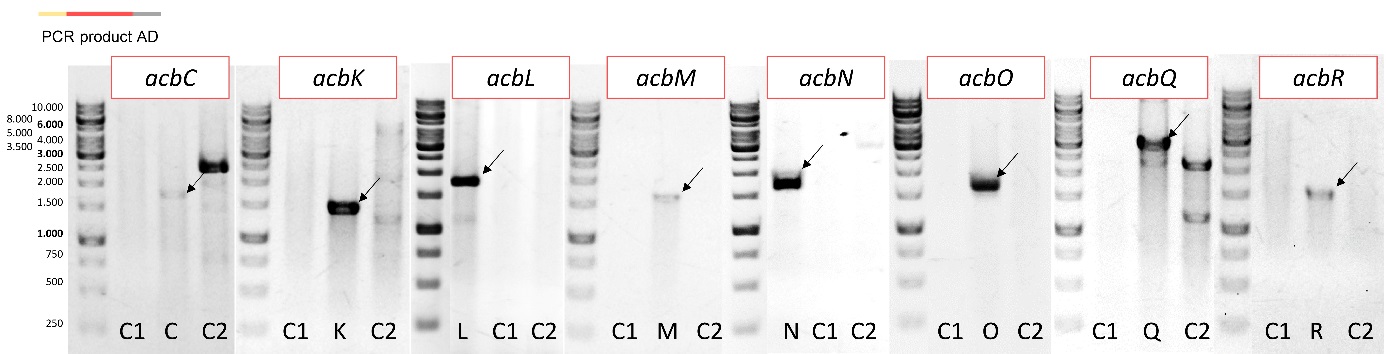


**Data S1.5.** Result of test-PCR shown for PCR-primer A and D displaying presence of cells with pKC1139-construct integrated into the genetic locus of the gene of interest (proof of vector integration in all cases).

## Data S2. Reduced transcription of *acb* genes downstream of the locus of vector integration shown by RT-qPCR for the mutant *Actinoplanes*sp. SE50/110 [pKC1139::P*_ermE_*_*_:*acbL*]*.*


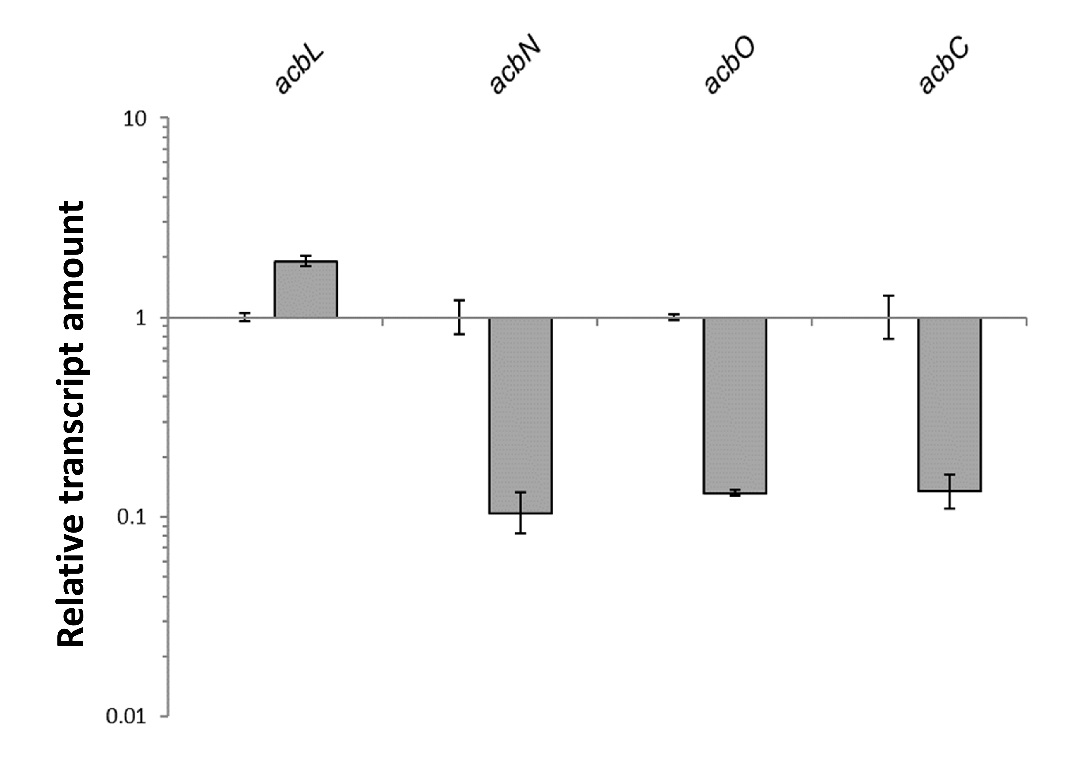


**Data S2.1.** Relative transcript amounts of *acbL* and *acb* genes down-stream of *acbL* in *Actinoplanes* sp. SE50/110 [pKC1139::*P_ermE_*_*_:*acbL*]. The RNA was isolated from the exponentially grown cultures of a shake flask cultivation in maltose minimal medium and analyzed by RT-qPCR. The transcript amounts were analyzed in relation to the wild-type. Shown are the means and standard deviation of at least three biological replicates. The RT-qPCR indicates significant increase of gene expression compared to the empty vector control (set to a value of 1) for the gene *acbL* and significant decrease of the downstream lying genes *acbN, acbO* and *acbC*. Significance was tested by a two-sided t-test (p-values: 0.0001123 (*acbL*), 0.0001535 (*acbN*), 2.65e-06 (*acbO*), 0.0006491 (*acbC*)).

## Data S3. Results of promoter screening experiment by use of the GUS-assay.


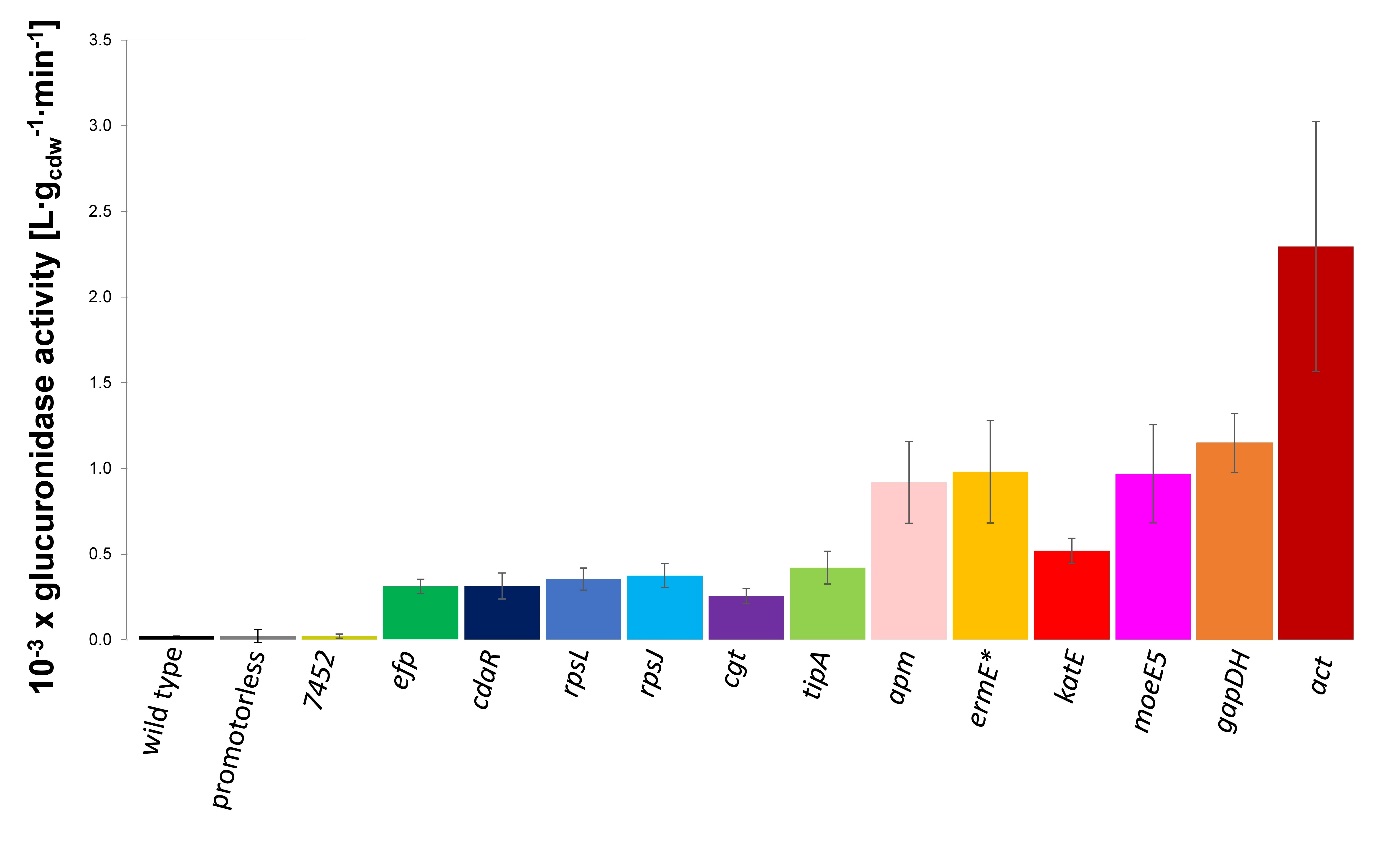


**Data S3.1.** Result of GUS-assay by use of entire cells washed in GUS-buffer: Glucuronidase activities, shown by the turnover of product (absorbance measured at 630 nm) over time normalized to the cell dry weight [g∙L^-1^] of three biological replicates (measured in 2 technical replicates).


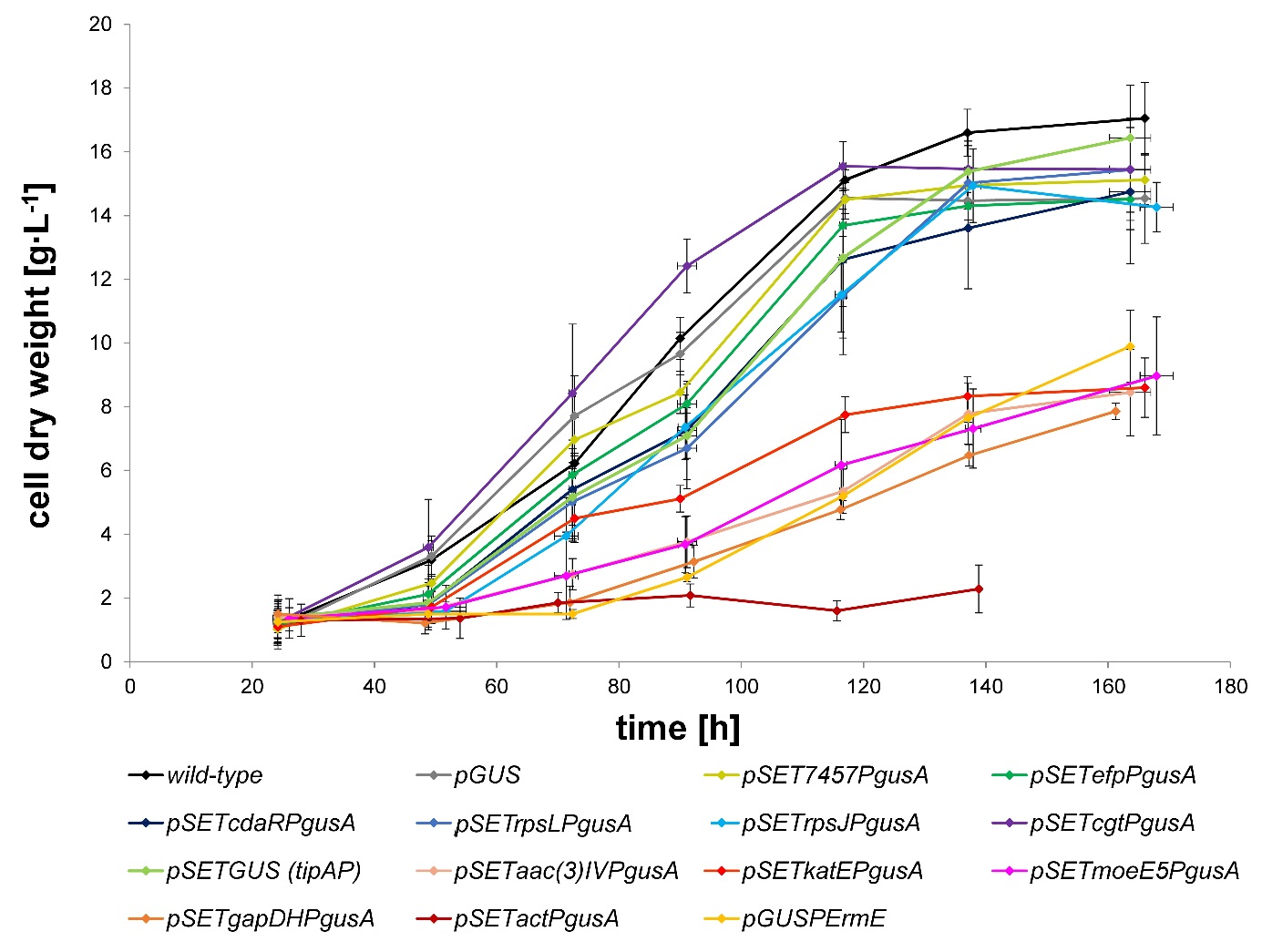


**Data S3.2.** Growth of *Actinoplanes* sp. SE50/110 in a shake flask cultivation in maltose minimal medium carrying different promoter constructs (**Table 1**). Shown are the cell dry weights [g∙L^‑1^]. Error bars on the x-axis display shift of sample time, when several cultivations were merged in on curve. (Number of biological replicates: wild-type: n = 3, pGUS: n = 6, pSET*7457*P*gusA*: n = 3, pSET*efp*P*gusA:* n = 5, pSET*cdaR*P*gusA*: n = 6, pSET*rpsL*P*gusA*: n = 6, pSET*rpsJ*P*gusA*: n = 4, pSET*cgt*P*gusA:* n = 6, pSETGUS (with *tipA*-promoter): n = 5, pSET*aac(3)IV*P*gusA:* n = 6, pSET*katE*P*gusA*: n = 3, pSET*moeE5*P*gusA*: n = 6, pSET*gapDH*P*gusA*: n = 4, pSET*act*P*gusA*: n = 3, pGUSPErmE: n = 2).

## Data S4. Determination of the transcription start sites of heterologous promoters in *Actinoplanes* sp. SE50/110 by 5‘-end specific transcriptome sequencing

The promoter structure influences binding and clearance of RNA polymerase and therefore substantially influences expression of a gene. A promoter usually consists of a -10 and a -35-region, an extended -10-motif and A+T-rich upstream promoter elements. Most of these elements are optional, whereas the -10-region is essential [59]. Knowledge about the transcription start sites (TSS) of genes allows genome-wide localization and determination of the promoter regions. In our group, a special protocol for the amplification of primary transcripts was developed, including the capture of primary transcripts, rewriting them into cDNA (complementary DNA) and amplification in the further course of the protocol [55].

Here, TSS were manually determined with special regard to the heterologous promoters. For each construct, at least one and up to three different TSS were found, leading to the identification of one or several -10-core-hexamers (**Data S4.1**). These were located mostly 6 to 7 nucleotides upstream of each TSS, which corresponds to the average distance of 6.4 nt described for *Actinoplanes* sp. SE50/110 by Schwientek *et al.* (2014) [60]. In accordance with recent results from *Corynebacterium glutamicum* [59], the comparison of these motifs point out, that especially the A at second and the T at last position are conserved, indicating, that they might be essential for promoter recognition and strength. The T on position 1 of the ‑10-hexamer shows the lowest conservation, similar to findings from *C. glutamicum* [59].

From previous studies it is known, that the -10-region ((A/T)ANNNT) is separated from the -35-region (TTGNNN) by a 16.6 nt spacer [60, 16]. Although only weekly conserved in *Actinoplanes* sp. SE50/110 [60], we could identify a -35-region in the range of the expected distance to the -10-region for most TSS (**Data S4.1**).

**Data S4.1.** Identified promoter motifs according to a primary transcript library. In case of several TSS upstream of the coding region, the corresponding promoters are enumerated by their proximity to the first nucleotide of the start codon (+1). The main promoter of each upstream region is highlighted in grey. Promoter structure: Bold letters indicate conserved nucleotides of the -35- and -10-hexamer. Additionally, the distance between both hexamers (s1) and the distance to the start codon (s2) are specified. If present, an extended -10-motif is written in small letters in front of the ‑10-hexamer.

|  | **promoter** | **promoter structure** | | | | |  |
| --- | --- | --- | --- | --- | --- | --- | --- |
|  |  | **-35** | **s1** | **-10** | **s2** | **+1** | |
| **homologous** | **Consensus motif according to Wolf et al. (2017)** | **TTG**NNN | **16.6** | **tg**n**TA**NNN**T** | **6.4** |  | |
|  | ***cgt-3*** | **T**GTCAT | **16** | **tg**gC**A**TT**CT** | **6** | **G** | |
|  | ***cgt-2*** | C**T**AAAT | **16** | **TA**GGC**T** | **6** | **G** | |
|  | ***cgt-1 (main)*** | **TTG**ACC | **17** | C**A**CTG**T** | **7** | **G** | |
|  |  |  |  |  |  |  | |
|  | ***efp*** | **TT**CGCC | **19** | c**g**g**TA**AAG**T** | **6** | **G** | |
|  |  |  |  |  |  |  | |
|  | ***rpsJ*** | **TT**AGCA | **18** | g**g**gC**A**TAC**T** | **6** | **G** | |
|  |  |  |  |  |  |  | |
|  | ***katE-2*** |  |  | G**A**TAC**T** | **6** | **G** | |
|  | ***katE-1 (main)*** | **TT**TGCC | **15** | g**g**g**TA**TCCG | **6** | **G** | |
|  |  |  |  |  |  |  | |
|  | ***7457-2*** | **TT**CCGT | **16** | **TA**CCG**T** | **8** | **A** | |
|  | ***7457-1 (main)*** | **TT**AGCT | **16** | **tg**a**TA**TCG**T** | **7** | **G** | |
|  |  |  |  |  |  |  | |
| **heterologous** | ***tipA-2*** | **T**ATCCC | **17** | C**A**CCTC | **6** | **C** | |
|  | ***tipA-1 (main)*** | **T**A**G**AAC | **16** | C**A**CGTC | **7** | **G** | |
|  |  |  |  |  |  |  | |
|  | ***moeE5-1 (main)*** | G**T**CGAG | **16** | G**A**ACG**T** | **5** | **G** | |
|  |  |  |  |  |  |  | |
|  | ***apm-3*** | **TTG**CAA | **15** | C**A**GAAA | **5** | **A** | |
|  | ***apm-2*** | **T**GCAAG | **17** | a**g**aA**A**AAT**T** | **6** | **A** | |
|  | ***apm-1 (main)*** | **TTG**CAA | **18** | **tg**c**TA**TGA**T** | **6** | **A** | |
|  |  |  |  |  |  |  | |
|  | ***cdaR-2*** | **TT**CGGC | **16** | C**A**ACT**T** | **7** | **G** | |
|  | ***cdaR-1* (main)** |  |  | G**A**CGCG | **7** | **C** | |
|  |  |  |  |  |  |  | |
|  | ***ermEP2*** |  |  | C**A**CGTG | **6** | **C** | |
|  |  |  |  |  |  |  | |
|  | ***ermEP1* ^1^*** | **T**G**G**GCA | **16** | **tg**g**TA**GGA**T** | **6** | **A** | |
|  |  |  |  |  |  |  | |
|  | ***gapDH-3*** | **TTG**CAG | **18** | c**g**c**TA**TGA**T** | **9** | **C** | |
|  | ***gapDH-2* (main)** | **T**G**G**GCG | **17** | c**g**gGGCGT**T** | **6** | **A** | |
|  | ***gapDH-1*** | **TT**CCTG | **16** | **TA**TCTG | **6** | **G** | |
|  |  |  |  |  |  |  | |
|  | ***rpsL-2 (main)*** | **T**GCTGT | **18** | A**A**TCCA | **6** | **G** | |
|  | ***rpsL-1*** | **T**CCACC | **17** | g**g**tCTCCG**T** | **7** | **A** | |

^1^Due to technical reasons, the TSS of *ermEP1**could not be detected in a mixed 5’-library. The promoter motif is based on sequence comparison and TSS detection by RNASeq in *Streptomyces lividans* TK23 performed by Siegl et al. [51].

We found extended -10-motifs consisting of a TG-dimer or a single G (**Data S4.1**), like described by Wolf *et al.* (2017) [16]. Extended -10-motifs mainly occur in Gram-positive bacteria and are assumed to enhance the promoter activity [61]. In the past, it has been suggested that the occurrence of this motif is associated with a poor conservation of the -10-sequence motif [61] or absence of a -35-hexamer [61, 62]. However, the influence of TG-dimers in *Actinoplanes* sp. SE50/110 is poorly analyzed yet. An enhancing function like in other bacteria is assumed.

In the promoters analyzed in this work, the first nucleotide of TSS is most often a purine, with G occurring more frequent than A (**Data S4.1**). In only three promoters, transcription starts on a C, similar to previous findings of Schwientek *et al.* (2014) [60].

Interestingly, the identified TSS and predicted promoter sequence of the *tipA*-promoter completely differs from the published one, which was identified in 1989 by S1-mapping in the host *S.* *lividans* [63]*.* Both – novel sequencing techniques with exact nucleotide-accuracy as well as the different host background – might explain this aberration. It also has to be noted, that the *tipA* promoter is constitutive in actinobacterial species, like f. e. *A. teichomyceticus* [19] and *Actinoplanes*sp. SE50/110 [23], whereas inducible by thiostrepton in *Streptomyces* [64, 19]. Here, transcription of the *tipA* promoter requires presence of TipAL [64].

A TipAL-homologue from *S. lividans*TK24 could not be identified in *Actinoplanes*sp. SE50/110 by BlastP-analysis (data not shown).

For the *ermE**-promoter, consisting of two parts (*ermEP2* and *ermEP1**) [51], only the TSS of *ermEP2* could be detected. For *ermEP1** TSS detection was not possible in a mixed sample due to technical reasons. Fortunately, TSS detection of *ermEP1** has already been performed with RNA-seq techniques by Siegl *et al* (2013) [51] in the host background of *S. lividans* TK23. These data were used to predict a promoter motif in *Actinoplanes* sp. SE50/110 (**Data S4.1**).

In summary, this library was used to identify promoter motifs of the main sigma factor σ^A^ according to Wolf *et al*. (2017) [65]. However, the influences of alternative sigma factors and other regulatory elements, like activators and repressors, are unknown.

In the case of the promoter of *moeE5,* pleiotropic regulation in the host species *S. ghanaensis* has already been reported [66]*.* Here, transcription of *moe* genes is directly influenced by the AraC-family transcriptional activator AdpA (*SSFG_04571*), which is conserved in *Streptomyces* [66].

In *S. coelicolor,* the promoter region of the activator of actinorhodin biosynthetic genes *actII-4* is positively influenced by an RNAse III homologue AbsB [67] and negatively influenced by the transcriptional regulator AtrA [68] and by the pleiotropic AbsA two-component signal transduction system [67]. The latter is probably also involved in negative regulation of the promoter region of the transcriptional activator *cdaR*, although in this case, the results of different studies are not completely consistent [46, 47, 69].

Also expression of the promoter regions of *ermE* and *rpsL*, which have been regarded as constitutive promoters, can vary during cultivation and might be therefore affected by pleiotropic effects during morphological differentiation [64].

Several putative gene homologues of the pleiotropic regulators mentioned above – with exception of AtrA – exist in the genome of *Actinoplanes* sp. SE50/110 according to BlastP analysis performed with the NCBI database (**Data S4.2**).

As the conservation of the promoter motif is one of several components allowing bacteria to regulate the strength of transcription, prediction of promoter motifs and/or transfer of strong promoters from related species are useful strategies to achieve overexpression, but do not entirely replace an individual promoter screening in the particular host due to versatile other regulatory effects.

**Data S4.2.** BlastP analysis in *Actinoplanes* sp. SE50/110.

| **Query** | **Subjects found in the genome of SE50/110 (GenBank: LT827010.1)** | **Identities and positives [%]** |
| --- | --- | --- |
| AraC-family transcriptional activator AdpA (*SSFG_04571*) from *S. ghanaensis* | *ACSP50_1149* | 48 % / 63 % |
|  | *ACSP50_1130* | 48 % / 69 % |
|  | *ACSP50_1044* | 48 % / 59 % |
|  | *ACSP50_7006* | 43 % / 59 % |
|  | *ACSP50_5299* | 47 % / 59 % |
|  | *ACSP50_7006* | 44 % / 58 % |
|  | *ACSP50_3870* | 42 % / 56 % |
|  | *ACSP50_2856* | 39 % / 52 % |
| two-component system transcriptional repressor AbsA2 (*SCO3226*) from *S. coelicolor* | *ACSP50_6187* | 48 % / 66 % |
|  | *ACSP50_6797* | 47 % / 66 % |
|  | *ACSP50_1881* | 48 % / 63 % |
|  | *ACSP50_7237* | 49 % / 62 % |
|  | *ACSP50_6876* | 48 % / 62 % |
|  | *ACSP50_2440* | 46 % / 64 % |
|  | *ACSP50_3776* | 46 % / 62 % |
|  | *ACSP50_5206* | 45 % / 59 % |
|  | *ACSP50_6859* | 42 % / 62 % |
|  | *ACSP50_5079* | 45 % / 63 % |
|  | *ACSP50_5197* | 44 % / 64 % |
|  | *ACSP50_4165* | 43 % / 60 % |
|  | *ACSP50_5324* | 43 % / 59 % |
|  | *ACSP50_3112* | 43 % / 60 % |
|  | *ACSP50_5286* | 47 % / 64 % |
|  | *ACSP50_1126* | 43 % / 62 % |
|  | *ACSP50_5600* | 42 % / 60 % |
|  | *ACSP50_1842* | 44 % / 60 % |
|  | *ACSP50_6565* | 41 % / 58 % |
| two-component system transcriptional repressor AbsB (*SCO5572*) from *S. coelicolor* | *ACSP50_7319* | 64 % / 75 % |
| two-component system transcriptional repressor AtrA (*SCO4118*) from *S. coelicolor* | *not found* | - |

## Data S5. Growth and acarbose formation of pSET152-based *acbC*-overexpression mutants.


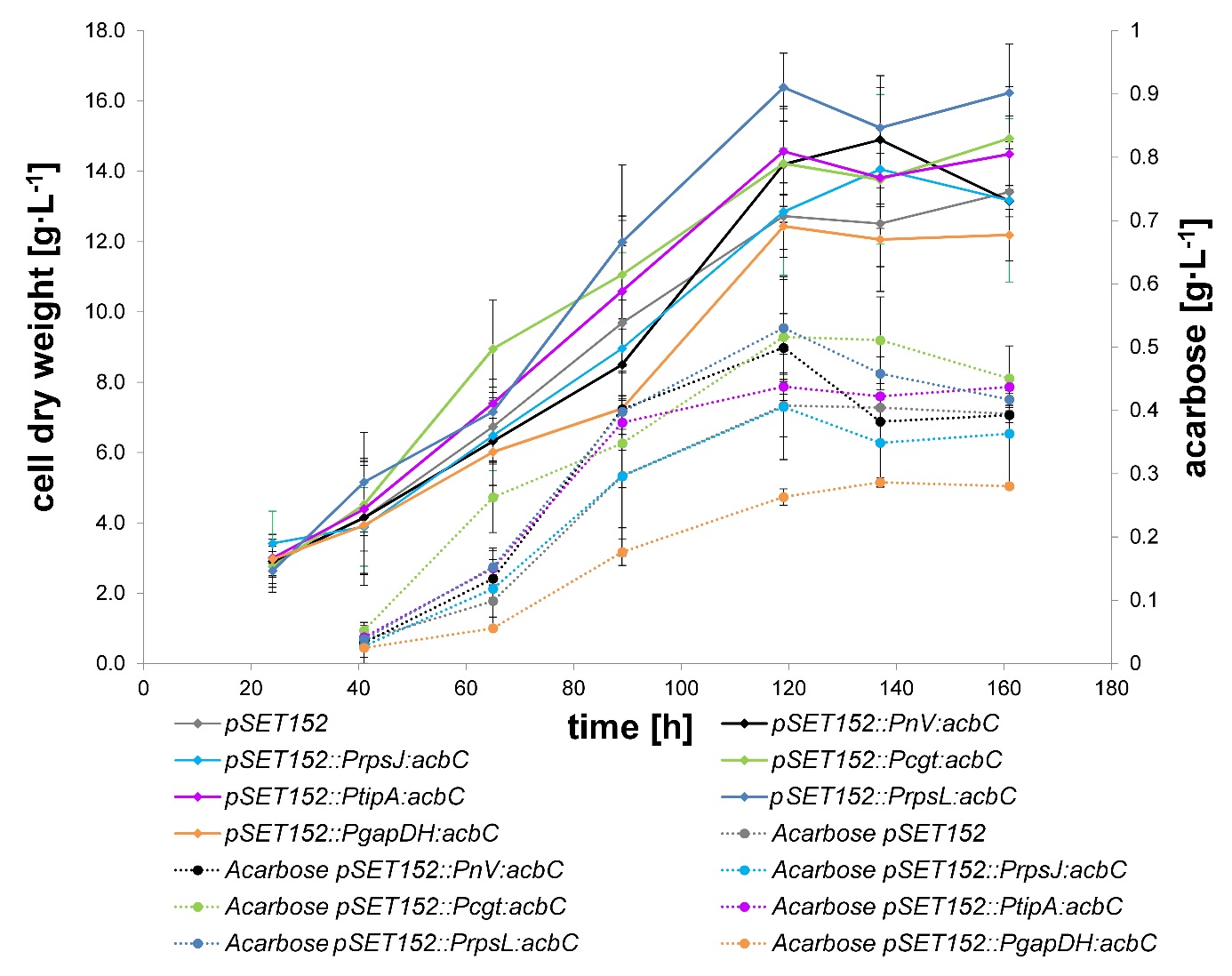


**Data S5.1.** Growth of *Actinoplanes* sp. SE50/110 in a shake flask cultivation in maltose minimal medium carrying different pSET152 based *acbC* overexpression mutants. Shown are the cell dry weights [g∙L^-1^] and acarbose concentration in the supernatant [g∙L^-1^]. (Number of biological replicates: pSET152: n = 3, pSET152::P*nV:acbC*: n = 3, pSET152::P*rpsJ:acbC*: n = 3, pSET152::P*cgt:acbC*: n = 4, pSET152::P*tipA:acbC*: n = 4, pSET152::P*rpsL(XC):acbC*: n = 4, pSET152::P*gapDH:acbC*: n = 5).

## Data S6. Smart formula analysis of the isotopic pattern of mass m/z = 255.03 [M-H^+^].

We performed SmartFormula analysis (Bruker Daltonik GmbH, Bremen, Germany), which generates a molecular formula from a specific mass. Three formulas were generated from the mass m/z = 255.03 [M-H^+^] by SmartFormula (**Data S6**). According to the sigma factor (mSigma), which reports the statistical variance between the measured and theoretical isotopic profile based on the intensity values of the peaks in the pattern, the formula C_7_H_12_O_8_P has got the best mScore. Formula and specific mass correspond to valienol-7-phosphate (M = 256.15 g/mol). The two remaining formula generated by SmartFormula can be excluded, as they either do not represent an intermediate known from bacteria (#2 of **Data S6**) or do not contain a phosphorus group (#3 of **Data S6**), which has already been shown by MS/MS for compound m/z = 255.03 [M-H^+^].

**Data S6.1**. Identified molecular formulas generated by SmartFormula (Bruker Daltonik GmbH) from the specific mass m/z = 255.03 [M-H]. Shown are the measured mass and the theoretical mass of the generated formulas [m/z] as well as the error [ppm]. The molecular formulas are sorted according the mSigma which reports the statistical variance between the measured and theoretical isotopic profile based on the intensity values of the peaks in the pattern.

| **Meas. m/z** | **#** | **Formulas** | **Calc m/z** | **err [ppm]** | **mSigma** |
| --- | --- | --- | --- | --- | --- |
| 255.0304 | 1 | C_7_H_12_O_8_P | 255.0275 | -11.3 | 10.9 |
|  | 2 | C_11_H_13_O_3_P_2_ | 255.0345 | 16.2 | 21.0 |
|  | 3 | C_14_H_7_O_5_ | 255.0299 | -2.0 | 40.3 |

**Additional References**

55. Pfeifer-Sancar K, Mentz A, Rückert C, Kalinowski J. Comprehensive analysis of the Corynebacterium glutamicum transcriptome using an improved RNAseq technique. BMC Genomics. 2013;14:888. doi:10.1186/1471-2164-14-888.

56. Bolger AM, Lohse M, Usadel B. Trimmomatic: a flexible trimmer for Illumina sequence data. Bioinformatics. 2014;30:2114–20. doi:10.1093/bioinformatics/btu170.

57. Langmead B, Salzberg SL. Fast gapped-read alignment with Bowtie 2. Nat Methods. 2012;9:357–9. doi:10.1038/nmeth.1923.

58. Hilker R, Stadermann KB, Schwengers O, Anisiforov E, Jaenicke S, Weisshaar B, et al. ReadXplorer 2-detailed read mapping analysis and visualization from one single source. Bioinformatics. 2016;32:3702–8. doi:10.1093/bioinformatics/btw541.

59. Albersmeier A, Pfeifer-Sancar K, Rückert C, Kalinowski J. Genome-wide determination of transcription start sites reveals new insights into promoter structures in the actinomycete Corynebacterium glutamicum. J Biotechnol. 2017;257:99–109. doi:10.1016/j.jbiotec.2017.04.008.

60. Schwientek P, Neshat A, Kalinowski J, Klein A, Rückert C, Schneiker-Bekel S, et al. Improving the genome annotation of the acarbose producer Actinoplanes sp. SE50/110 by sequencing enriched 5'-ends of primary transcripts. J Biotechnol. 2014;190:85–95. doi:10.1016/j.jbiotec.2014.03.013.

61. Vašicová P, Pátek M, Nešvera J, Sahm H, Eikmanns B. Analysis of the Corynebacterium glutamicum dapA Promoter. J Bacteriol. 1999;181:6188–91.

62. Kumar A, Malloch RA, Fujita N, Smillie DA, Ishihama A, Hayward RS. The minus 35-recognition region of Escherichia coli sigma 70 is inessential for initiation of transcription at an "extended minus 10" promoter. J Mol Biol. 1993;232:406–18. doi:10.1006/jmbi.1993.1400.

63. Murakami T, Holt TG, Thompson CJ. Thiostrepton-induced gene expression in Streptomyces lividans. J Bacteriol. 1989;171:1459–66.

64. Myronovskyi M, Luzhetskyy A. Native and engineered promoters in natural product discovery. Nat Prod Rep. 2016;33:1006–19. doi:10.1039/c6np00002a.

65. Wolf T. Transcriptional regulation of acarbose biosynthesis in Actinoplanes sp. SE50/110 analyzed by next-generation sequencing, transcriptomics and genome editing; 2017.

66. Makitrynskyy R, Ostash B, Tsypik O, Rebets Y, Doud E, Meredith T, et al. Pleiotropic regulatory genes bldA, adpA and absB are implicated in production of phosphoglycolipid antibiotic moenomycin. Open Biol. 2013;3:130121. doi:10.1098/rsob.130121.

67. Aceti DJ, Champness WC. Transcriptional Regulation of Streptomyces coelicolor Pathway-Specific Antibiotic Regulators by the absA and absB Loci. J Bacteriol. 1998;180:3100–6.

68. Uguru GC, Stephens KE, Stead JA, Towle JE, Baumberg S, McDowall KJ. Transcriptional activation of the pathway-specific regulator of the actinorhodin biosynthetic genes in Streptomyces coelicolor. Mol Microbiol. 2005;58:131–50. doi:10.1111/j.1365-2958.2005.04817.x.

69. Sheeler NL, MacMillan SV, Nodwell JR. Biochemical activities of the absA two-component system of Streptomyces coelicolor. J Bacteriol. 2005;187:687–96. doi:10.1128/JB.187.2.687-696.2005.
